# Redistribution of Histone Marks on Inflammatory Genes Associated With Intracerebral Hemorrhage-Induced Acute Brain Injury in Aging Rats

**DOI:** 10.3389/fnins.2022.639656

**Published:** 2022-04-15

**Authors:** Qin Zhang, Wei-lin Kong, Jun-Jie Yuan, Qiong Chen, Chang-Xiong Gong, Liang Liu, Fa-Xiang Wang, Jia-Cheng Huang, Guo-Qiang Yang, Kai Zhou, Rui Xu, Xiao-Yi Xiong, Qing-Wu Yang

**Affiliations:** ^1^Department of Neurology, Xinqiao Hospital, The Army Medical University (Third Military Medical University), Chongqing, China; ^2^Acupuncture and Tuina School, Third Teaching Hospital, Chengdu University of Traditional Chinese Medicine, Chengdu, China; ^3^Acupuncture and Chronobiology Key Laboratory of Sichuan, Chengdu, China

**Keywords:** hemorrhage, neuroinflammation, histones, acute, aging

## Abstract

The contribution of histone mark redistribution to the age-induced decline of endogenous neuroprotection remains unclear. In this study, we used an intracerebral hemorrhage (ICH)-induced acute brain injury rat model to study the transcriptional and chromatin responses in 13- and 22-month-old rats. Transcriptome analysis (RNA-seq) revealed that the expression of neuroinflammation-associated genes was systematically upregulated in ICH rat brains, irrespective of age. Further, we found that interferon-γ (IFN-γ) response genes were activated in both 13- and 22-month-old rats. Anti-IFN-γ treatment markedly reduced ICH-induced acute brain injury in 22-month-old rats. At the chromatin level, ICH induced the redistribution of histone modifications in the promoter regions, especially H3K4me3 and H3K27me3, in neuroinflammation-associated genes in 13- and 22-month-old rats, respectively. Moreover, ICH-induced histone mark redistribution and gene expression were found to be correlated. Collectively, these findings demonstrate that histone modifications related to gene expression are extensively regulated in 13- and 22-month-old rats and that anti-IFN-γ is effective for ICH treatment, highlighting the potential of developing therapies targeting histone modifications to cure age-related diseases, including brain injury and neuroinflammation.

## Introduction

Intracerebral hemorrhage (ICH)-induced acute brain injury leads to high mortality and disability in patients, and approximately half of them do not survive beyond few months of ICH onset, while most survivors suffer permanent function loss (Mayer and Rincon, 2005; Flower and Smith, 2011; An et al., 2017). Aging is a major risk factor for ICH (Saloheimo et al., 2001; Gong et al., 2004; Ruiz-Sandoval et al., 2006; Lee et al., 2009; Okauchi et al., 2009, 2010; van Asch et al., 2010; Partridge, 2014). Compared with young animals, ICH-induced acute brain injuries demonstrate inferior lesion resolution, more severe edema, and poorer function recovery in aging animals (Gong et al., 2004, 2011; Lee et al., 2006; Wasserman et al., 2008; Lively and Schlichter, 2012). The molecular processes by which aging predisposes animal brains to ICH, or the mechanisms of deregulation in ICH, remain to be determined.

Inflammation is a key response of the natural defense mechanism against ICH-induced secondary brain injury (Zhou et al., 2014; Chen et al., 2015). Intriguingly, both neurodegenerative diseases and normal aging lead to immune activation in the brain (Lucin and Wyss-Coray, 2009; Barrientos et al., 2015; Chen et al., 2016). We speculate that age-regulated inflammatory and immune responses may play crucial roles in determining the outcome and treatment of ICH.

Epigenetic alteration is a known hallmark of aging (Fraga and Esteller, 2007; Lopez-Otin et al., 2013). Mounting evidence demonstrates that aging and neurodegenerative diseases are accompanied by changes in histone modifications (Lardenoije et al., 2015; Booth and Brunet, 2016; Sen et al., 2016). For example, DNA methylation decreases with age, and its distribution patterns are altered by age and environmental factors in a tissue-specific manner (Wilson and Jones, 1983; Bollati et al., 2009; Christensen et al., 2009). Age-related DNA methylation changes in brains have been identified and shown to have a strong association with Alzheimer's disease (Horvath et al., 2012; Numata et al., 2012; De Jager et al., 2014). On the one hand, the activation of histone 3 lysine 4 trimethylation (H3K4me3) and repression of H3K27me3 are epigenetic modifications that are closely associated with transcription and directly involved in lifespan regulation in multiple organisms. The gain of both activating and repressing marks is characteristic of aging in worms (Jin et al., 2011; Maures et al., 2011; Sen et al., 2016), but not in flies (Li et al., 2010; Ni et al., 2012). On the other hand, histone acetylation is generally associated with transcription activation and exhibits an age-dependent global increase in yeast, worm, flies, and human cells (Scalabrino and Ferioli, 1984; Struhl, 1998; Eisenberg et al., 2009; Peleg et al., 2016; Pradeepa et al., 2016). Additionally, histone modifications are involved in regulating the expression of immune/inflammatory response genes and have recently been shown to be involved in regulating innate immune memory (Tabas and Glass, 2013; Falkenberg and Johnstone, 2014; Ivashkiv and Donlin, 2014; Netea et al., 2015, 2016; Daskalaki et al., 2018; Natoli and Ostuni, 2019).

Therefore, in this study, we hypothesized that age-regulated histone modifications in the brain may be involved in the age-dependent inflammatory response and brain injury during ICH and may be useful for developing novel ICH therapeutics.

## Materials and Methods

### Animals

In this study, 13-month-old (middle age) and 22-month-old (late age) male Sprague Dawley rats were used. The rats were obtained from the Animal Center of Daping Hospital, the Army Medical University (the Third Military Medical University, Chongqing, China), and housed in specific pathogen-free conditions. The rats were randomly selected in each experimental group. Animal studies were approved by the Animal Ethics Committee of the Army Medical University (Third Military Medical University, Chongqing, China) and conducted in accordance with their guidelines.

### ICH Models

We established a rat ICH model by inducing ICH in the striatum (caudate + putamen), according to the method of Ni et al. (2015). Briefly, rats were anesthetized with 3.5 and 1.5% isoflurane for induction and maintenance, respectively, and then immobilized on a stereotaxic apparatus (RWD Life Science Co., Shenzhen, China), followed by the drilling of a 1-mm diameter burr hole, 0.2 mm anterior and 3.5 mm lateral to the bregma. We then injected a total of 75 μl of autologous blood into the striatum using a Hamilton needle attached to a micropump (RWD Life Science Co.), 5.5 mm ventral to the skull surface, as described in our previous study (Yuan et al., 2019).

### Western Blotting

Total cellular protein was extracted, and Western blotting was performed, as described in our previous study (Xiong et al., 2016b). Briefly, protein samples from the ipsilateral brain tissues were resolved in sodium dodecyl sulfate-polyacrylamide gel electrophoresis (SDS-PAGE) gels and transferred onto polyvinylidene fluoride membranes by electroblotting. For Western blotting, the membranes were blocked with 5% skimmed milk in phosphate-buffered saline (PBS), then incubated with anti-H3K27me3 (Cell Signaling Technology, Danvers, MA, USA) at a dilution of 1:1,000, anti-H3K4me3 (Cell Signaling Technology) at a dilution of 1:1,000, anti-H3K9ac (Cell Signaling Technology) at a dilution of 1:1,000, and anti-GAPDH antibody (Abcam, Cambridge, MA, USA) at a dilution of 1:2,000. Thereafter, the membranes were incubated with a horseradish peroxidase-conjugated secondary antibody (Sigma-Aldrich, St. Louis, MO, USA) at a dilution of 1:2,500. The blots were visualized using the enhanced chemiluminescence system from ProteinSimple (San Jose, CA, USA) and normalized to GAPDH levels.

### Immunohistochemistry

Paraffin-embedded brain tissue sections were prepared, then deparaffinized in xylene and rehydrated in a series of graded ethanol solutions, as described in our previous study (Xiong et al., 2016b). The sections were then blocked with 3% hydrogen peroxide for 10 min at room temperature to abolish potential endogenous peroxidase activity, followed by antigen retrieval in 10 mmol/L citrate buffer at pH 6.0 for 15 min at 100°C. Next, the sections were incubated in 5% bovine serum albumin for 30 min to block nonspecific binding of the secondary antibody, followed by overnight incubation with anti-H3K27me3, H3K4me3, or H3k9ac antibody at a dilution of 1:100 (Cell Signaling Technology, USA) at 4°C. On the following day, the sections were washed with PBS three times and then incubated with horseradish peroxidase-conjugated goat anti-rabbit immunoglobulin G (IgG) (Boster, Beijing, China) at a dilution of 1:500 for 1 h at 37°C. Subsequently, the immunostained tissue sections were analyzed using an image analysis software (ImageJ, version 1.46J from National Institutes of Health, Bethesda, MD, USA). All immunostaining experiments were performed at the same time under the same conditions.

### Analysis of Brain Water Content

As reported in our previous study, the rats were anesthetized and sacrificed 3 days after ICH, and perihematomal brain tissues were obtained for analysis of the BWC (Xiong et al., 2016b). The resected brain tissues were wiped with filter paper to remove the water on their surfaces and subsequently weighed on an electronic balance to determine their wet weights. Thereafter, the brain tissues were dried at 100°C for 24 h, and the dry weights of the tissue samples were measured. The BWC was calculated using the following formula: BWC (%) = (wet weight–dry weight)/wet weight × 100%.

### Neurological Deficit Score

According to the methods reported in a previous study (Chen et al., 2001), rat NDS was assessed on days 1, 3, 5, and 7 post-ICH. Scoring was performed by three well-trained investigators who were blinded to all experimental data.

### Hematoxylin and Eosin Staining

Brain tissues were obtained from euthanized rats and fixed in 10% neutral-buffered formalin solution; then, they were dehydrated in ethanol solutions and embedded in paraffin. Next, tissue samples (5-μm thick) were prepared using a rotary microtome, then stained with H&E, and examined under a Leica microscope.

### Fluoro-Jade B Staining

According to the methods described in our previous study (Wang et al., 2017), we performed FJB staining to assess degenerative neurons. Briefly, brain tissues were dehydrated in 15 and 30% sucrose solutions, and then cut into 25-μm thick sections. The sections were then immersed in 1% sodium hydroxide in 80% alcohol for 10 min, in 70% alcohol for 2 min, and finally in distilled water for 5 min. Next, the sections were transferred into a 0.06% potassium permanganate solution and incubated for 10 min at room temperature, then washed with distilled water. Thereafter, the sections were incubated in a 0.01% FJB solution (Millipore, Massachusetts, USA) for 30 min and washed with distilled water, and then dehydrated in gradient alcohol solutions and cleared in xylene, and finally covered with coverslips in DPX (a non-aqueous, non-fluorescent, plastic mounting medium, Sigma-Aldrich). The FJB-stained tissue samples were independently examined and quantified in each standardized microscopic field by three researchers using ImageJ (version 1.46J, National Institutes of Health).

### Terminal Deoxynucleotidyl Transferase dUTP Nick End Labeling and Nissl Staining

Terminal deoxynucleotidyl transferase dUTP nick end labeling (TUNEL) staining was performed using a commercial In Situ Cell Death Detection Kit (Roche, Basel, Switzerland) according to the manufacturer's instructions. Briefly, a 50-μl TUNEL reaction mixture was added to the brain tissue slices, which were then incubated at 37°C for 1 h and washed with Tris-buffered saline three times; the negative control was prepared by adding 50 μl of marking solution to the brain tissue slices. Next, the slices were incubated with 50–100 μl of 3,3′-diaminobenzidine (DAB) solution or POD substitute solution for 10 min, then dehydrated with gradient alcohol solutions, cleared in xylene, and covered with coverslips in neutral gum (Sigma-Aldrich).

For Nissl staining, brain tissue slices were stained with thionin solution (Sigma-Aldrich), dehydrated in alcohol, cleared in xylene, and covered with coverslips. Subsequently, the regions of the ipsilateral and contralateral brain tissues were analyzed. The TUNEL- and Nissl-stained tissue sections were analyzed under a Leica microscope. All quantitative histological analyses were performed in each standardized microscopic field (100 μm) of three brain slices. TUNEL and Nissl staining-positive cell counts of the brain tissue were performed by three researchers using ImageJ (version 1.46J, National Institutes of Health, Bethesda, MD, USA). These data are shown as the mean value of cells per section, based on the average number of cells in three sections.

### Construction of RNA-Seq Library and DNA Sequencing

To construct an RNA-seq library, total cellular RNA was extracted from brain tissue samples using TRIzol reagent (Ambion, Austin, TX, USA) according to the manufacturer's instructions and then treated with RQ1 DNase (Promega, Madison, WI, USA) to remove DNA; the extracted RNA was quantified by measuring the absorbance at 260/280 nm using Smartspec plus (Bio-Rad, Hercules, CA, USA). The integrity of the extracted RNA was further assessed by 1.5% agarose gel electrophoresis. Thereafter, 10 μg of each total RNA sample was used to construct the RNA-seq library. Specifically, polyadenylated mRNA samples were isolated, purified, and concentrated with oligo (dT)-conjugated magnetic beads (Invitrogen, Carlsbad, CA, USA), fragmented at 95°C, and ligated using end repair and 5' adaptor. The mRNA samples were then reverse-transcribed into cDNA using the RT primer harboring the 3'-end adaptor sequence and a randomized hexamer, which was purified and amplified using PCR, leading to the generation of 200–500 bp PCR products. The cDNA was purified, quantified, and stored at −80°C until DNA sequencing was performed. High-throughput DNA sequencing of the cDNA libraries was performed using the Illumina NextSeq 500 system for 150-nt paired-end sequencing analysis by ABlife Inc. (Wuhan, China), according to the manufacturer's instructions.

### RNA-Seq Data Analysis

To analyze the RNA-seq data, raw DNA reads with more than 2-N base reads, clipping adaptor, low-quality bases (less than 20), and markedly short reads (less than 16 nt) were discarded using the Cutadapt software, as described previously (Martin, 2011). Then, the reads were compared with the Rattus norvegicus genome sequence (Rnor 6.0) and annotation file (Rnor 6.0.82) in the Ensembl database (http://asia.ensembl.org/Rattus_norvegicus/Info/Index) by aligning to the rat genome using TopHat2 with the end-to-end method allowing two mismatches, as described previously (Kim et al., 2013). Aligned DNA reads with more than one genomic localization were also discarded, and uniquely localized reads were analyzed for reads number and RPKM value (reads per kilobase and per million). We then determined the gene coverage and depth, read distribution around transcription start sites (TSSs), and transcription terminal sites (TTSs).

After obtaining the gene expression data of all samples, we identified differentially expressed genes (DEGs) using the R package edgeR, according to the method of Robinson et al. (2010). Moreover, the adjacent time point samples were compared to determine the genes regulated by age, and the treated and normal ICH samples at the same time points were also compared. For each gene, we obtained the *p*-value using the negative binomial distribution model and estimated the fold change using this package when the *p*-value < 0.01 and 2-fold change was achieved.

### Genomic Locus Clustering Analysis of DEGs

After identifying the DEGs, we clustered them according to their genomic loci and chromosomal location and calculated the average distance (AD) for the DEGs in each chromosome using the formula of AD between adjacent genes (AD = chromosome length/DEG number). Adjacent genes with real distance < AD were clustered together.

### Reverse Transcription-Quantitative Polymerase Chain Reaction

We performed RT-PCR to confirm the presence of selected DEGs in tissue samples using the primers listed in Supplementary Table 4. RNA samples were reverse transcribed into cDNA using the PrimeScriptTM RT Reagent Kit (Takara, Dalian, China), which was in turn amplified using the ChamQTM SYBR Color qPCR Master Mix (Vazyme, Nanjing, China), and the expression levels were normalized to GAPDH levels. RT-PCR was performed in triplicate for different tissue samples.

### ChIP Library Construction and Sequencing

Frozen tissues were first gently rinsed with 40 ml of deionized Milli-Q water two times, fixed in 37 ml of 1.0% formaldehyde, and then vacuum-filtered for 10 min using a desiccator attached to a vacuum pump. Next, the tissue samples were quenched and cross-linked by adding 2.5 ml of 2 M glycine to achieve a final concentration of 0.125 M, followed by vacuum-filtration for an additional 5 min, then rinsed two times with Milli-Q water and dried to remove water from the samples. The tissue samples were then ground in liquid nitrogen to obtain a fine powder using mortar and pestle. Then, 1 ml of nuclei lysis buffer [50 mM Tris-HCl (pH 8.0), 10 mM EDTA (pH 8.0), and 1% sodium dodecyl sulfate] was added to the powdered tissue; the mixture was vortexed and placed on ice for extracting chromatin, then sonicated to obtain 100- to 500-bp DNA fragments. The samples were centrifuged in a microfuge at 10,000 rpm at 4°C for 10 min. Subsequently, we transferred 4 × 60-μl aliquots of chromatin samples into separate siliconized microfuge tubes, added 540 μl of ChIP dilution buffer (1% Triton X-100, 1.2 mM EDTA, 167 mM NaCl, 16.7 mM Tris-HCl at pH 8.0) into each tube, and added the appropriate antibody into each tube. The antibodies used in this study were anti-H3K27me3 (Millipore, Cat #07-449), anti-H3K4me3 (Millipore, Cat #17-614), anti-H3K9ac (Millipore, Cat #06-942), and anti-rabbit IgG (Millipore, Cat #12-370). The mixtures were then incubated on a rotating mixer at 4°C overnight. To isolate the immune complex formed therein, 50 μl of protein A Dynabeads was added to the mixtures and rotated at 4°C for 2 h before removing the supernatants. Specifically, after the addition of the magnetic beads into the tube, the mixtures were incubated for 5 min with gentle rotation at 4°C after adding 1 ml of each of the selected buffers, i.e., one time with a low-salt wash buffer (150 mM NaCl, 20 mM Tris-HCl at pH 8.0, 0.1% SDS, 0.5% Triton X-100, and 2 mM EDTA), a high-salt wash buffer (500 mM NaCl, 20 mM Tris-HCl at pH 8.0, 0.1% SDS, 0.5% Triton X-100, and 2 mM EDTA), and the LiCl wash buffer (0.25 M LiCl, 1% sodium deoxycholate, 10 mM Tris-HCl at pH 8.0, 1% NP-40, and 1 mM EDTA), and two times with TE buffer (1 mM EDTA and 10 mM Tris-HCl at pH 8.0). The immunoprecipitated samples were eluted from the magnetic beads using the elution buffer [50 nM Tris (pH 8.0), 10 mM EDTA, and 1% SDS] and reverse cross-linked by overnight incubation at 65°C, then subjected to sequential RNase A and proteinase K treatment, and purified by phenol extraction and ethanol precipitation. Finally, high-throughput DNA sequencing of the prepared libraries was performed using the ThruPLEX DNA-seq 48S Kit according to the manufacturer's instructions (Cat #R400427, Rubicon Genomics, Ann Arbor, USA) in Illumina NextSeq 500 System by ABlife Inc. (Wuhan, China).

### ChIP-Seq Data Analysis

After obtaining the DNA sequences, we first removed adaptors and low-quality bases from the raw reads using Cutadapt (Martin, 2011) and aligned the sequences to the rat-Rnor6.0 genome using bowtie2 software (Langmead and Salzberg, 2012). We then combined the replicate samples during the peak-calling step because of the high correlation coefficients between replicates. Next, we enriched and identified the peaks of the immunoprecipitates over input experiments using MACS2 v2.0 and selected peaks with more 4-fold enrichment as candidate peaks. For each gene, the adjacent 5,000 bp from the TSS was defined as the promoter region using bedtools (v2.20.1). Peak-associated genes identified specifically in Ipsi or control samples were regarded as peak-associated differentially modified genes (pDMGs) for each histone mark in each month.

To analyze the reads-associated DMGs (rDMGs), we followed the ChIP-seq analysis protocol reported by Nativio et al. (2018). Briefly, we merged peaks from each sample into one peak if their genomic loci overlapped. We then regarded the number of reads in each merged peak as its peak density. The peak density was normalized using a per-sample reads-per-million (RPKM) scalar coefficient and the size of the region of analysis (in kb). Adjusted peak density was averaged over two replicating samples of the same group, and the input was subtracted, thus obtaining the histone mark enrichment value or area under the curve. The log value of normalized density after subtracting the input was used as the input value for determining the statistical significance of the differential modified peaks by performing Student's *t*-test for two-way comparisons (Ipsi vs. cont, 22-month-old rats vs. 13-month-old rats, *p*-value <0.05). For peak-associated genes, the adjacent 3,000 bp around the TSS of each gene was the associated peak region. Genes with peak in this region were regarded as histone mark genes, and genes with significantly modified peaks were regarded as rDMGs.

### ChIP-qPCR

We performed qPCR to identify the genes using the same protocol as for the ChIP-seq experiments. Anti-H3K27me3, anti-H3K4me3, or anti-H3K9ac antibody, and control IgG (all from Millipore) were used for the ChIP assay. qPCR using SYBR green real-time PCR mixture (11202ES08, Yeasen, Shanghai, China) in a real-time detection system (Bio-Rad) was performed for the ChIP samples, with the primers listed in Supplementary Table 4.

### Statistical Analysis

Analyses were performed using the SPSS 13.0 software, and the values are presented as mean ± SEM. Statistical significance was calculated using Student's *t*-test for comparison between data from two groups. Two-way ANOVA with repeated measures was performed when appropriate to compare the data from repeated measurements. All statistical analyses of RNA-seq and ChIP-seq data were performed using the R software (https://www.r-project.org/), unless otherwise indicated. The K-S test was used to determine the distribution profile difference, hypergeometric test was used for creating the Venn diagram, and Gene Ontology (GO) and Kyoto Encyclopedia of Genes and Genomes (KEGG) pathway and network analyses were performed to analyze functional term enrichment. ^*^p < 0.05, ^**^p < 0.01, and ^***^p < 0.001 indicate different statistical significance levels. Hierarchical clustering analysis was performed using Cluster 3.0 or heatmap function in R.

## Results

### Brains of 13-Month-Old Rats Are Less Damaged Than Those of 22-Month-Old Rats After ICH

To study the dynamics of ICH-induced neuroinflammation and histone modifications in aging rats, we evaluated the responses of 13- and 22-month-old rats, which roughly correspond to 40- and 60-year-old human, respectively, to ICH treatment. In our ICH model, autologous blood was injected into the left brains of rats (Supplementary Figure 1A).

**Figure 1:**
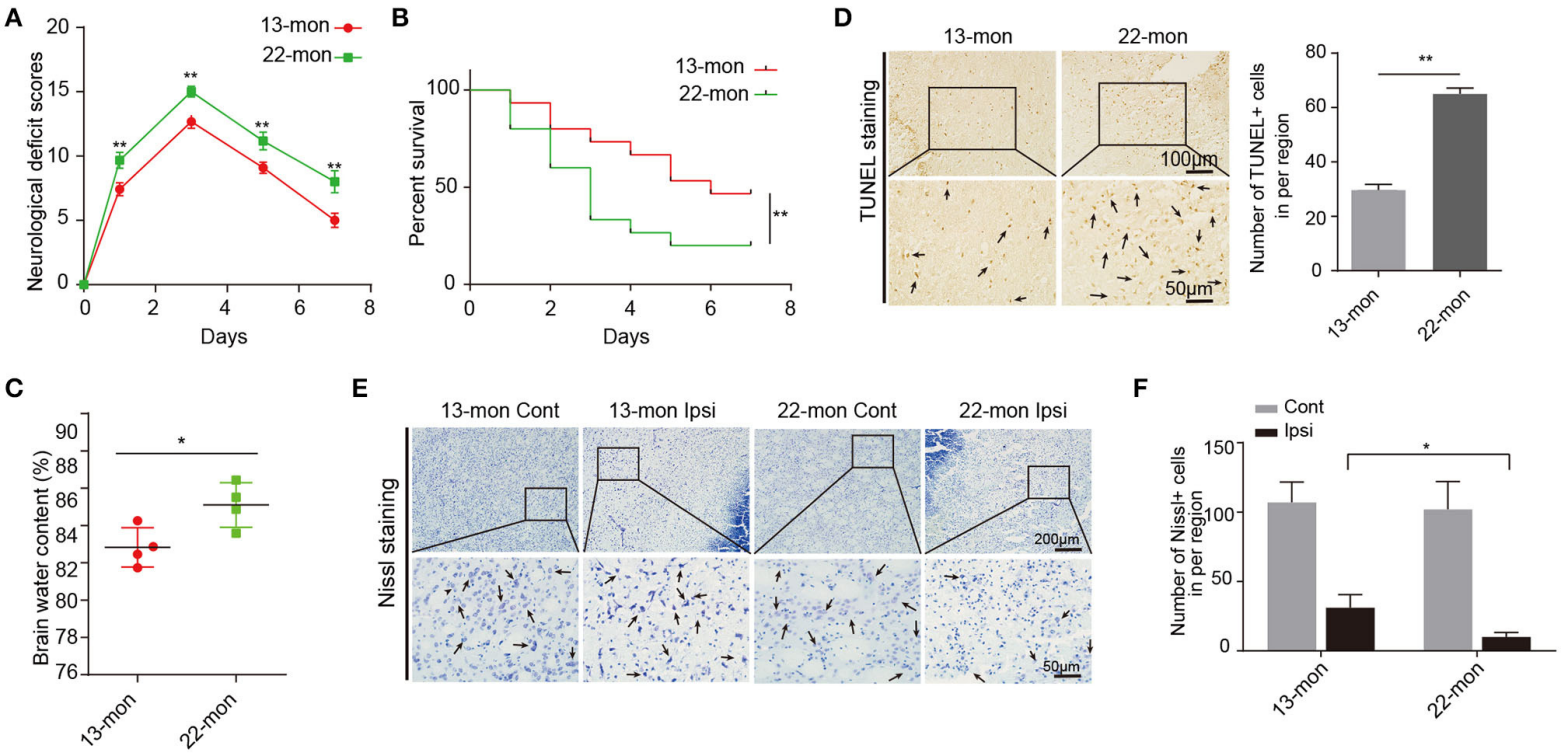
Acute brain injury and mortality induced by ICH in rats. **(A)** The neurological deficit scores of 13- and 22-month-old ICH rats (*n* = 12). **(B)** The survival rates of 13- and 22-month-old ICH rats (*n* = 15). **(C)** Level of brain water contents in perihematomal brain tissues in 13- and 22-month-old rats 3 days after ICH surgery (*n* = 4). **(D)** TUNEL assay and quantification of apoptotic neural cells in perihematomal brain tissues derived from 13- and 22-month-old rats 3 days after ICH surgery. Scale bars, 100 μm at the top and 50 μm at the bottom. **(E,F)** Nissl staining and quantification of Nissl bodies in perihematomal brain tissues derived from 13- and 22-month-old rats 3 days after ICH surgery. Scale bars, 200 μm at the top and 50 μm at the bottom. The bar graphs show the mean ± SEM. *p*-value was determined by using the Student's *t*-test. *p < 0.05 and **p < 0.01, respectively.

Compared with 13-month-old rats, 22-month-old rats showed more pronounced ICH damage, as reflected by multiple parameters including the NDS, BWC, survival rates, FJB staining, and TUNEL staining (Figures 1A–D and Supplementary Figures 1B,C). Neuron survival was significantly lower in the ipsilateral brain than in the contralateral hemispheres (Figures 1E,F). Notably, 13-month-old rat brains exhibited significantly less brain damage after ICH than 22-month-old rat brains (Figure 1 and Supplementary Figure 1).

### Expression of Neuroinflammation-Associated Genes in Brain Was Similarly Upregulated After ICH in Both 13- and 22-Month-Old Rats

After 3 days of ICH, contralateral and ipsilateral brain tissues were collected for RNA-seq analysis. Two independent benches of experiments, including three sets of experimental repeats, were performed to validate the results of transcriptome analysis. Over 100 million reads for each tissue sample were obtained (Supplementary Table 1). Among these, 77.76–84.00% were matched against the rat genome, with ~90% showing a unique genomic locus. The genomic region (Supplementary Figure 2A) and transcript distribution (Supplementary Figure 2B) of the aligned reads were well balanced (Mortazavi et al., 2008).

**Figure 2:**
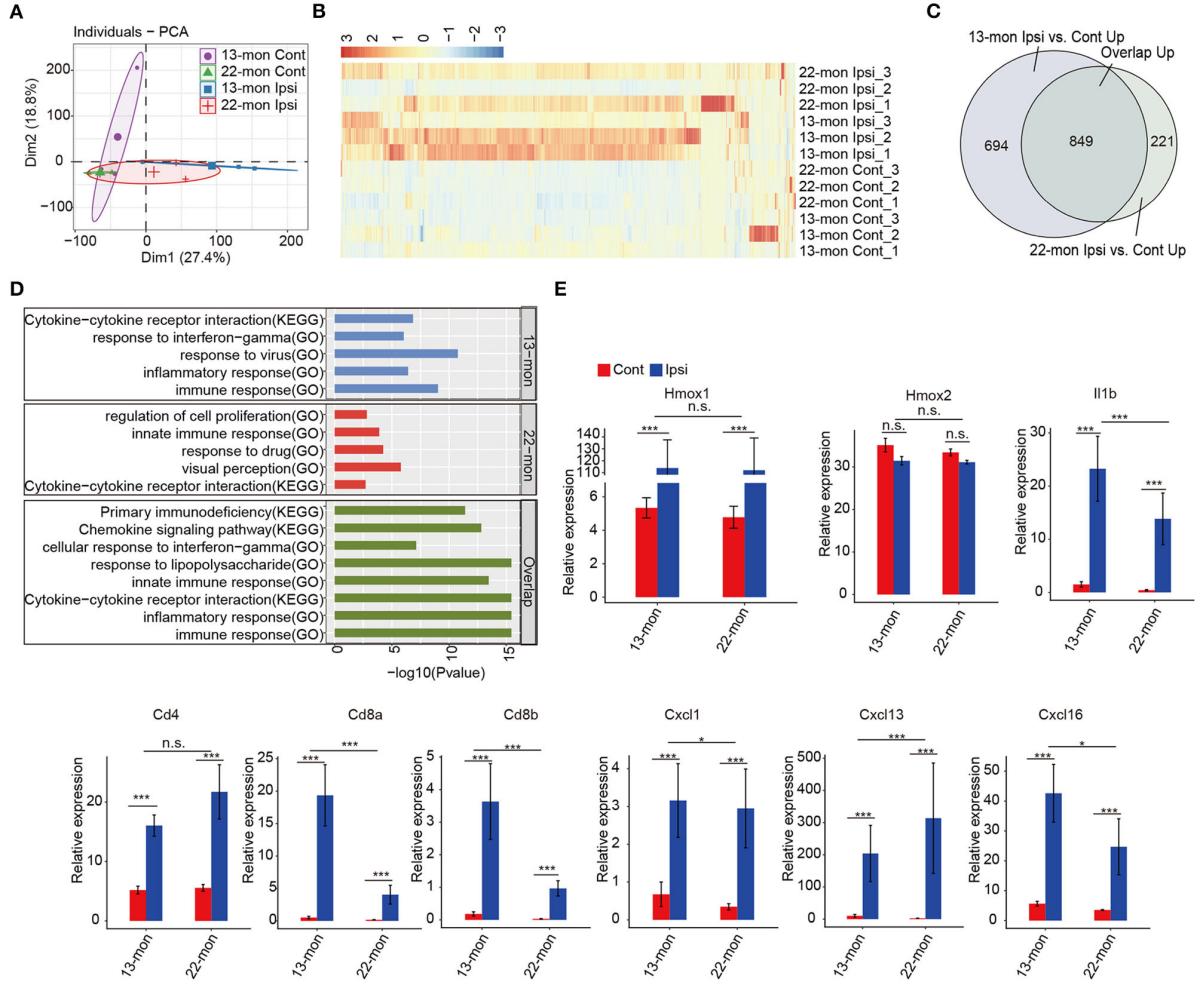
Enrichment of inflammation and immune response-associated genes in ICH-induced acute brain injury of aging ICH rats. **(A)** Principal component analysis shows distinct divergence between the ipsilateral and contralateral brain tissues of RNA-seq samples. **(B)** Hierarchical clustering heat map showed the dominant upregulation of differentially expressed genes (DEGs) between the ipsilateral and contralateral brain tissues of aging rats 3 days after ICH surgery. **(C)** Venn diagram shows the highly overlapped, upregulated DEGs between 13- and 22-month-old ICH rats. **(D)** Bar plot shows the top enriched GO terms and KEGG pathways of specifically upregulated DEGs in 13-month-old (top panel) and 22-month-old ICH rats (middle panel), and overlapped, upregulated DEGs between 13- and 22-month-old rats (bottom panel). **(E)** Bar plot shows the expression levels of genes related to the inflammatory response. The bar graphs show the mean ± SEM; *p*-value was determined by using the Student's *t*-test. *p < 0.05 and ***p < 0.001, respectively.

Principal component analysis revealed that the gene expression profiles in ipsilateral brain samples differed from those in the contralateral brain samples (Figure 2A). Analysis of the DEGs revealed upregulation rather than downregulation of gene expression in ipsilateral brain tissues compared with that in contralateral brain tissues in both 13- and 22-month-old rat samples (Figure 2B). Additionally, genes with upregulated expression in 13- and 22-month-old rat brains significantly overlapped (*p* = 0, analyzed by the hypergeometric test; Figure 2C). Functional analysis of the upregulated genes revealed that they were highly enriched in the immune response, inflammatory response, and innate immune response pathways (Supplementary Figures 2C,D). The transcriptome analysis results of this study were consistent with those reported in previous studies (Zhou et al., 2014; Xiong et al., 2016a; Lan et al., 2017; Zhang et al., 2017), confirming that the expression of the immune/innate immune and inflammatory response pathways systematically increased in our ICH aging rat models. We further analyzed the functions of coregulated and specifically regulated DEGs in 13- and 22-month-old rat brain samples. Enrichment analysis demonstrated that the immune and inflammatory response pathways were highly enriched in three terms (Figure 2D). IFN-γ was enriched in 13-m-specific and co-upregulated genes (Figure 2D). Specifically, the expression of *Hmox1* gene, which encodes heme oxygenase-1 after ICH (Figure 2E) and is mainly induced in microglia, was remarkably induced (Matz et al., 1997; Nakaso et al., 2000). The expression of the pro-inflammatory cytokine *Il1b* significantly increased (Figure 2E). ICH-induced expression of *Cd4* marker in T helper cells was observed. In addition to the known cytokine *Cxcl1* marker of the infiltrating leukocytes, we identified a pronounced induction of *Cxcl13* and *Cxcl16* (Figure 2E).

### The Levels of H3K27me3, H3K4me3, and H3K9ac Modifications Indicate Dynamic Responses to Aging and ICH

**Figure 3:**
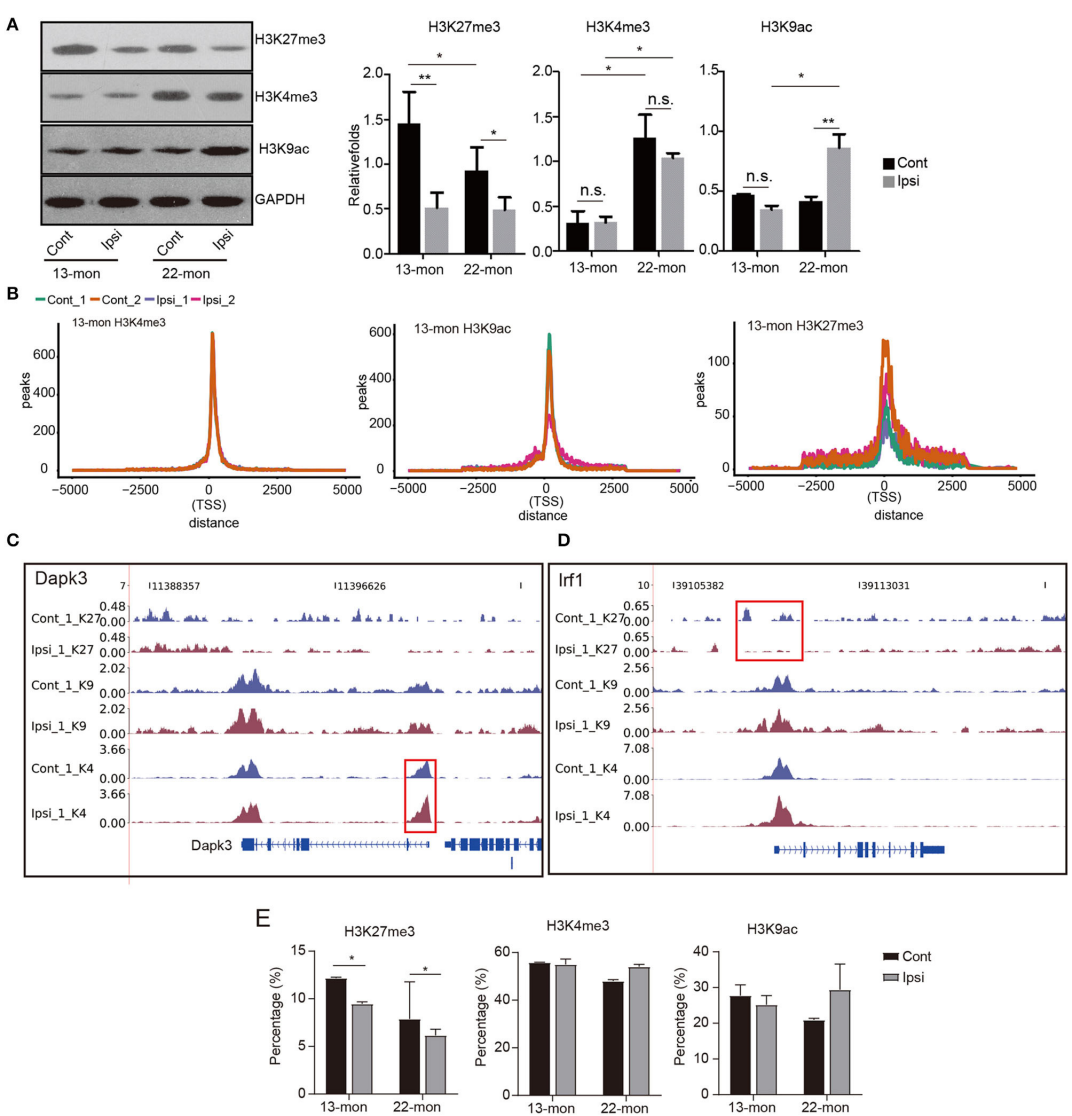
Global histone mark profile assessment reveals their distribution change in response to ICH treatment. **(A)** Western blotting and quantitative analysis of H3K27me3, H3K4me3, and H3K9ac in the ipsilateral and contralateral brain tissues of 13- and 22-month-old rats 3 days after ICH (*n* = 3). The bar graphs show the mean ± SEM. **(B)** Line plot shows the cumulative distribution of all uniquely mapped reads located within 5 kb around the transcription start sites (TSSs) of the three histone marks. ChIP-seq samples of Ipsi and control obtained from 13-month-old rats are shown in the figure. **(C,D)** Read density plot shows the distribution of three histone mark densities around the selected genes (*Dapk3, Irf1*, and *Il1b*). Gene structures are shown on the bottom track. ChIP-seq samples (the first replicate) of Ipsi and control from 13-month-old rats are shown in the figure. **(E)** Bar plot shows the percentage of peaks located within 2.5 kb around the TSS of the three histone marks. The bar graphs show the mean ± SEM. *p*-value in this figure was determined by using the Student's *t-*test. *p < 0.05 and **p < 0.01, respectively.

Transcriptional upregulation of genes involved in inflammatory and immune responses was highly similar in both 13- and 22-month-old rat brains. However, it was evident that 13- and 22-month-old rats differed significantly in their capabilities to respond to ICH damage (Figure 1 and Supplementary Figure 1). Thus, we aimed to determine whether the dynamics of histone marks were potential determinants of the age-dependent responses to ICH. According to the methods reported in previous studies, we selected the histones H3K27me3, H3K4me3, and H3K9ac as representatives to identify the age and ICH-related diseases (Kouzarides, 2007; Sen et al., 2016). We analyzed the levels of these three modifications in ipsilateral and contralateral brain tissues. Immunohistochemical and Western blot analyses showed that the levels of the repressive histone mark H3K27me3 significantly decreased, while the levels of the activating histone mark H3K4me3 were significantly upregulated in response to aging (Figure 3A and Supplementary Figure 3A). Additionally, H3K27me3 levels decreased in both ages of rats after ICH, while H3K9ac levels were significantly upregulated only in 22-month-old rats after ICH; H3K4me3 levels had no pronounced change in both ages of rats after ICH (Figure 3A and Supplementary Figure 3A). These results indicate different dynamics of the histone modification networks in 13- and 22-month-old ICH rat brains.

### ICH-Induced Redistribution of H3K27me3, H3K4me3, and H3K9ac in TSS With Aging

To further explore age- and ICH-induced changes in the epigenome, we performed ChIP-seq analysis using antibodies against H3K27me3, H3K4me3, and H3K9ac for the same tissues used in RNA-seq analysis. We obtained 48 datasets that included two replicates for each biological sample, half of which included the input controls, with over 34.65 × 106 (the mean of 54.59 × 106) uniquely aligned reads obtained from each ChIP-seq experiment (Supplementary Table 1), as recommended by the Encyclopedia of DNA Elements (ENCODE) guidelines (Landt et al., 2012). Genomic region distribution of aligned reads showed good consistency among ChIP-seq repeats of the same histone mark, and their predominant distribution in the intergenic regions was observed as expected (Supplementary Figures 3B,C). Peaks were observed either from each single ChIP-seq dataset or from both replicates using the MACS2 software (Zhang et al., 2008) and assigned to the promoter regions of genes within a defined distance to the TSS. Peaks from each single ChIP-seq dataset showed strong overlaps between replicates, further supporting the high quality of the datasets (Supplementary Table 2). Irreproducible discovery rate (IDR) analysis confirmed the quality of peaks recovered from the two replicates (Supplementary Figure 3D). The difference in peak quality scores also reflected the characteristics of histone mark deposition profiles, as described below.

The ChIP-seq peak signals of all three histone marks were found to be around the TSS (Figure 3B). The distribution of H3K4me3 peak reads in TSS was most clustered, while that of H3K27me3 peak signals was least clustered and had a saddle shape; this general feature was altered neither by age nor by ICH treatment (Figure 3B). Notably, the distributions of the H3K4me3 and H3K27me3 peak reads in TSS for each gene showed responses to environmental changes, as exemplified by *Dapk3* and *Irf1*, respectively (Figures 3C,D). *Dapk1* and *Stat1* also showed altered distribution in the promoter region (Supplementary Figure 3E).

Statistical analysis revealed that most TSS peaks were H3K4me3 peaks, followed by H3K9ac and H3K27me3 peaks (Figure 3E). Importantly, we showed that the H3K27me3 peaks in TSS significantly decreased after ICH in 13- and 22-month-old rat brains, while the H3K9ac peaks in TSS increased in 22-month-old rat brains (Figures 3A,E). Additionally, the H3K4me3 peaks in TSS decreased with age (Figure 3E). These results suggested that the redistribution of histone marks around TSS specifically responded to age and ICH, highlighting the importance of histone modifications in age-related ICH response.

### ICH-Induced Redistribution of H3K4me3 and H3K27me3 in the Promoter for Selected Neuroinflammation-Associated Genes in 13- and 22-Month-Old Rats, Respectively

**Figure 4:**
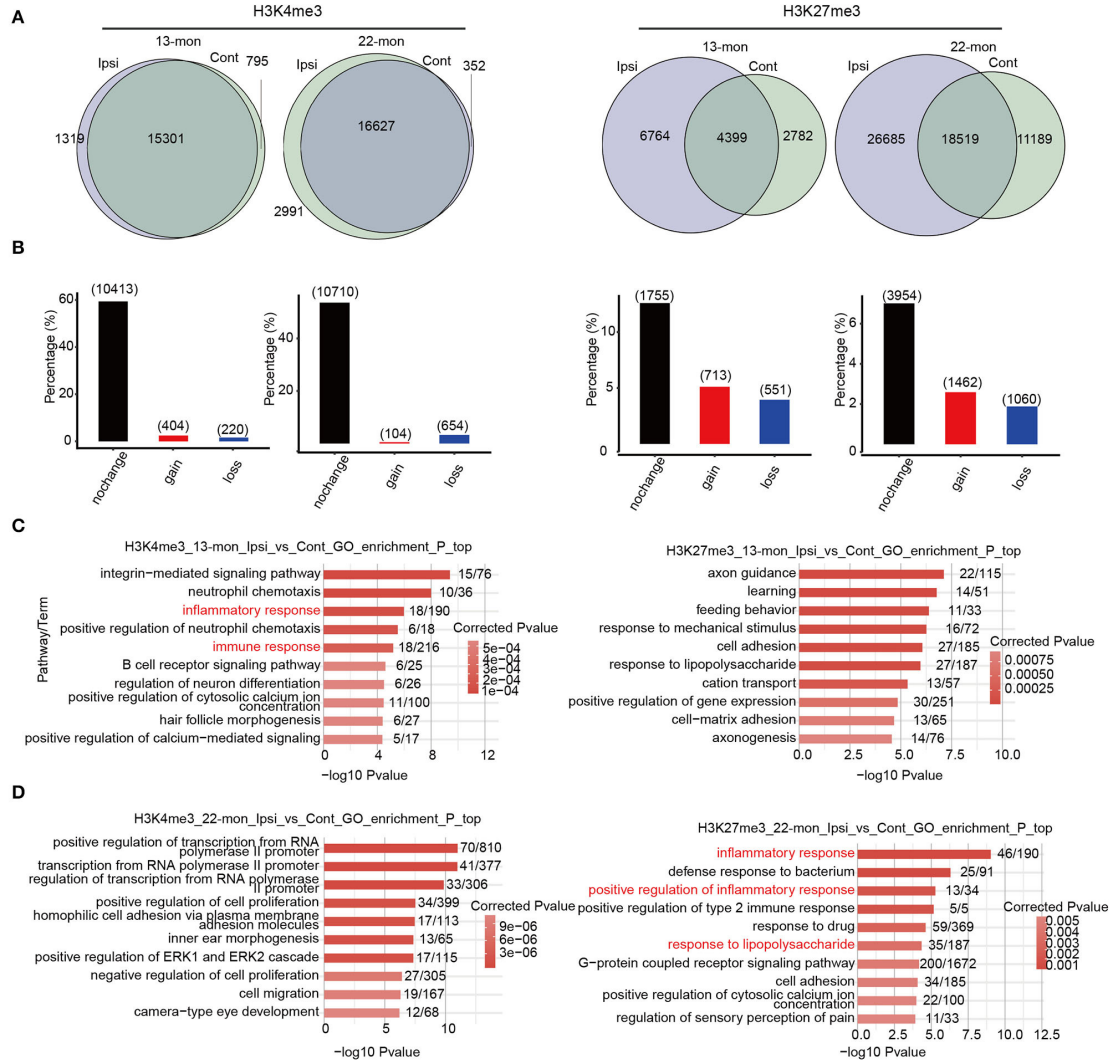
The distribution change of histone marks between Ipsi and control reveals their relevance to immune response genes. **(A)** Venn diagram shows the number of overlapped and specific peak clusters and genes (in the parentheses) between Ipsi and control samples for H3K4me3 and H3K27me3 in 13-month-old (left) and 22-month-old rats (right). **(B)** Bar plot shows the number of overlapped and specific genes between Ipsi and control samples for H3K4me3 and H3K27me3 in 13-month-old (left) and 22-month-old rats (right). **(C)** Bar plot shows the top 10 enriched GO BP terms for pDMGs with H3K4me3 (left) and H3K27me3 (right) marks in 13-month-old ICH rats. **(D)** Bar plot shows the top 10 enriched GO BP terms for pDMGs with H3K4me3 (left) and H3K27me3 (right) marks in 22-month-old ICH rats.

To investigate the potential contribution of the age- and ICH-regulated distributions of these three histone marks, we compared their peaks between each pair of ipsilateral and contralateral brain tissues to identify the peaks gained and lost after ICH (Figure 4A and Supplementary Figure 4A). Genes containing such peaks within 3 kb of the TSS sites (promoter regions) were selected. PDMG overlap analysis revealed that H3K4me3 peak containing genes were least affected by ICH, while H3K27me3 peaks were the most affected (Figure 4B and Supplementary Figure 4B). Further, we performed functional enrichment analysis for pDMGs. In 13-month-old rat brains, ICH-induced pDMGs containing the gain or loss of promoter-associated H3K4me3 mark were highly enriched in inflammatory and immune response functions (Figure 4C, left). However, no inflammatory and immune response functions were enriched by ICH-induced H3K27me3 and H3K9ac-associated pDMGs (Figure 4C, right and Supplementary Figure 4C). Strikingly, in 22-month-old rat brains, ICH-induced H3K27me3 pDMGs were remarkably enriched in inflammatory and immune response functions (Figure 4D, right), while those of H3K4me3 and H3K9Ac pDMGs were enriched in other functions (Figure 4D, left and Supplementary Figure 4D). Analysis of the functional terms enriched by the genes bearing unchanged peaks as controls revealed that the three histone marks selected in this study were distinct in their target function and did not exhibit remarkable differences between 13- and 22-month-old rat brains (Supplementary Figure 4E).

### ICH-Induced Histone Mark Redistribution and Gene Expression Are Correlated

**Figure 5:**
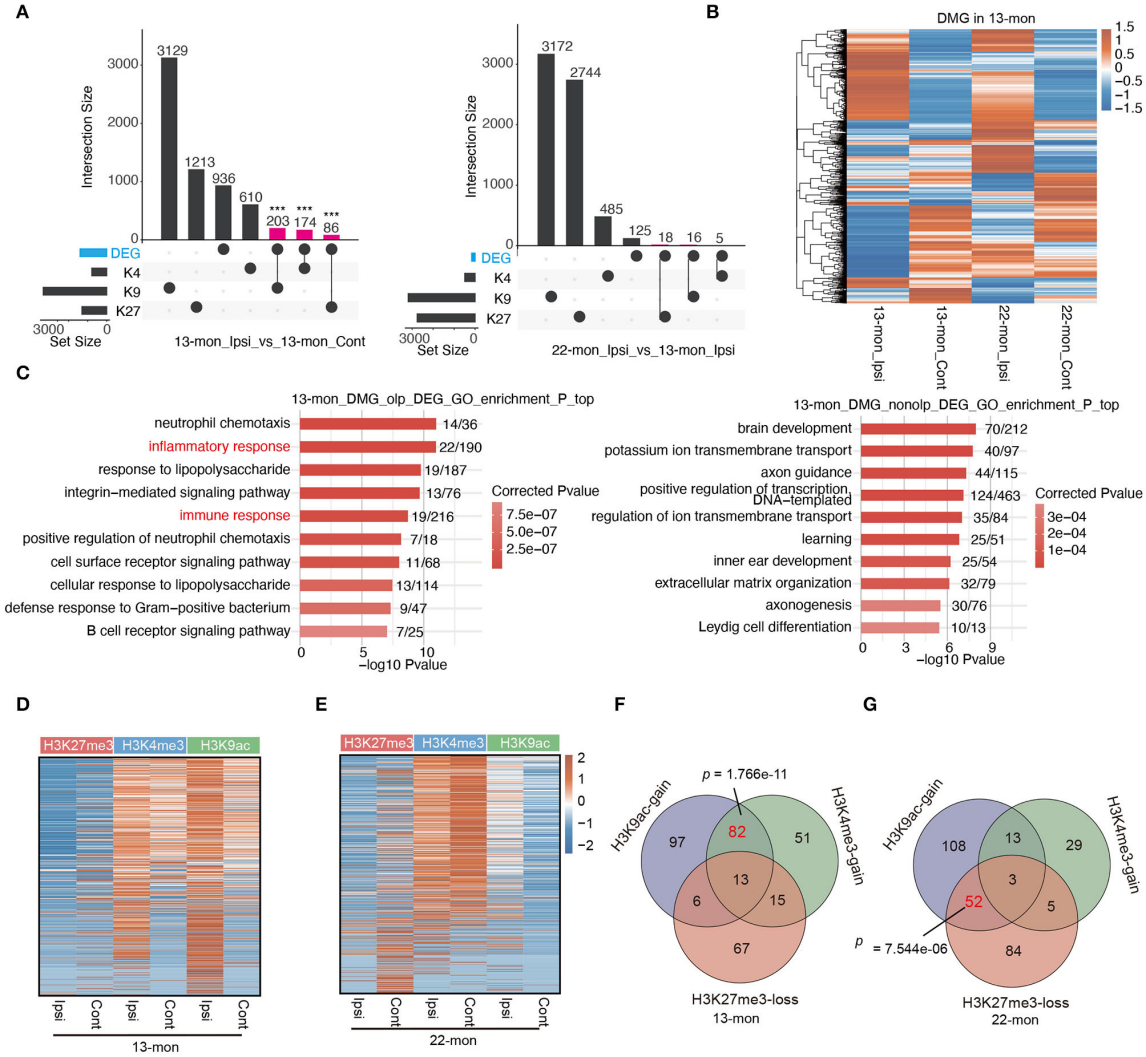
pDMGs significantly overlap with DEGs. **(A)** Upset bar plot shows the overlapped gene number among DEGs and three histone-modified genes in 13-month-old (left) and 22-month-old (right) Ipsi rats. **(B)** Hierarchical clustering heat map shows the expression level of genes that are differentially modified by histone marks in 13-month-old Ipsi rats. **(C)** Bar plot shows the top 10 enriched GO BP terms of DEGs that overlap with DMGs (left) or do not overlap with DMGs (right) in 13-month-old ICH rats. **(D)** Heatmap of the three histone marker signals at the TSS regions in 13-month-old rats for co-upregulated DEGs. The genes were ranked according to average fold enrichment using the MACS2 software. **(E)** Heatmap of the three histone marker signals at the TSS regions in 22-month-old rats for co-upregulated DEGs. The genes were ranked according to average fold enrichment using the MACS2 software. **(F)** Venn diagram shows the overlapping upregulated DEGs that are specifically enriched with H3K9ac, H3K4me3 markers, or shows the loss of H3K27me3 marker in 13-month-old ICH rats. **(G)** Venn diagram shows the overlapping upregulated DEGs that are specifically enriched with H3K9ac, H3K4me3 markers, or shows the loss of H3K27me3 marker in 22-month-old ICH rats. *P*-value was determined by using the Student's t-test. ***p < 0.001.

To explore the correlation between the pDMGs of each mark and the overall gene expression, we first overlapped the pDMGs with the DEGs identified earlier in this study and found a strong correlation between ICH-induced pDMGs and DEGs, when compared with those induced by age (Figure 5A and Supplementary Figure 5A). These results suggested that ICH-induced pDMGs are specifically associated with gene expression regulation. Heat map analysis of the expression levels of ICH-induced pDMGs further supported this speculation (Figure 5B and Supplementary Figure 5B). The DEG-overlapped pDMGs were more enriched in immune and inflammatory response functions when compared with the non-overlapped ones (Figure 5C and Supplementary Figure 5C), suggesting that ICH-induced alteration of the histone-associated chromatin structure was associated with the expression of neuroinflammation-associated genes.

We further explored the association between ICH-induced histone mark distribution and gene expression by plotting peak reads of all three histone marks in the TSS regions of all upregulated DEGs, thereby demonstrating that the TSS-associated histone marks changed in response to ICH in DEGs (Figure 5D). It is noteworthy that the dynamics of histone marks in 13- and 22-month-old rats differed significantly (Figures 5D,E). In 13-month-old rats, approximately half of the DEGs had relatively high levels of both H3K4me3 and H3K9ac in contralateral brain tissues, and ICH-induced increase in the levels of these histone marks was evident for almost all DEGs (Figure 5D). Indeed, a large fraction of the upregulated genes (95/331, 28.7%) was associated with the gain of both H3K4me3 and H3K9ac marks (Figure 5F). In contrast, the decrease of H3K27me3 mark in ICH-responsive DEGs was remarkably less compared with the increase of H3K4me3 and H3K9ac in 13-month-old rat brains (Figures 5D,F).

Transcription start sites-associated histone marks of ICH-responsive DEGs in 22-month-old rats were different from those observed in 13-month-old rats; ICH-induced loss of H3K27me3 and increase of H3K9ac were prevalent in most DEGs (Figures 5E,G). In summary, the association between DEGs and pDMGs containing differentially modified (DM) peaks of H3K4me3 and H3K9ac was more pronounced in 13-month-old rats than in 22-month-old rats (*p* = 1.766e-11, using Fisher's exact test; Figure 5F); the association between DEGs with pDMG containing DM peaks of H3K27me3 and H3K9ac was more pronounced in 22-month-old rat brains than in 13-month-old rat brains (*p* = 7.544e-06, using Fisher's exact test; Figure 5G).

### Increased Expression of IFN-γ Response Genes After Acute Brain Injury in 22-Month-Old ICH Rats

**Figure 6:**
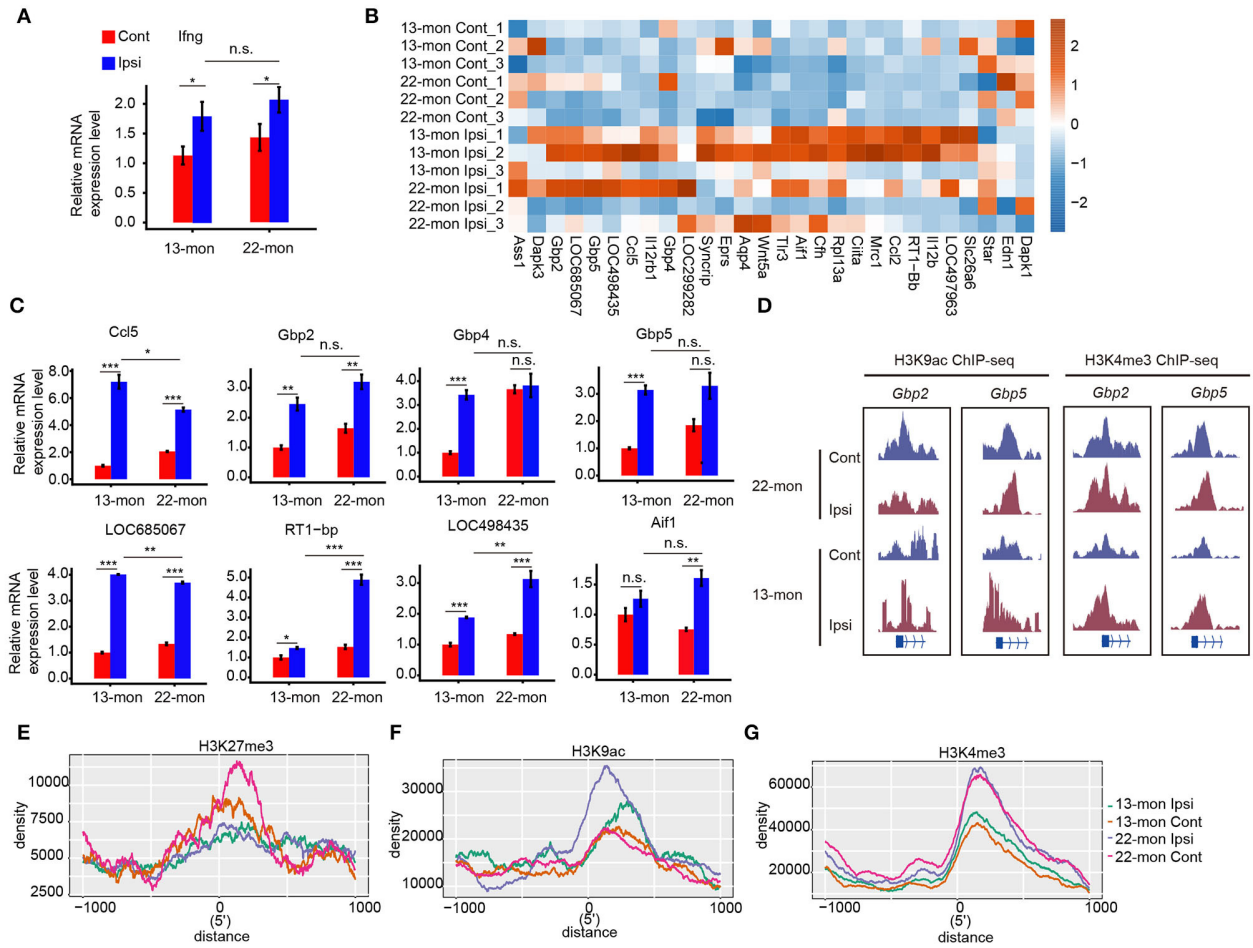
IFN-γ pathway regulated by histone modification mediates acute brain injury in aging ICH rats. **(A)** Bar plot shows the increased expression level of *Ifng* in 13- and 22-month-old ICH rats 3 days after ICH surgery by RT-qPCR. **(B)** Heat map shows the upregulation of DEGs in response to IFN-**γ** after ICH surgery. Color bar indicates the normalized expression level. **(C)** Bar plot shows the increased expression level of genes **(B)** in 13- and 22-month-old ICH rats 3 days after ICH surgery by RT-qPCR analysis. **(D)** Read density plot shows the H3K9ac and H3K4me3 ChIP-seq signals on *Gbp2* and *Gbp5*, respectively. The signal height indicates the read density per million total mapped reads. **(E–G)** Line plot shows the histone mark density around the TSS region of genes in response to IFN-γ. Three histone marks are shown separately. The bar graphs show the mean ± SEM; *p*-value was determined by using the Student's t-test. *p < 0.05, **p < 0.01 and ***p < 0.001, respectively.

The correlation between histone mark redistribution and gene expression prompted us to explore the potential contribution of histone mark redistribution on the ICH response pathway. We observed that the responses to IFN-γ were considerably upregulated in 13- and 22-month-old rat brain tissues (Figure 2D). qRT-PCR analysis showed that IFN-γ expression was significantly upregulated in 22-month-old ICH rat brains (Figure 6A). We then analyzed the IFN-γ response genes in the GO database by plotting the heatmap of their expression patterns, which revealed differential expression of these IFN-γ response genes in the brain samples of 22-month-old rats with or without ICH (Figures 6B,C). High levels of H3K9ac and/or H3K4me3 modifications were observed at the promoter regions of IFN-γ response genes (e.g., *Gbp2* and *Gbp5*) in aging ICH rats (Figure 6D). Analysis of histone mark distribution around the TSS regions of all IFN-γ response genes showed that the H3K27me3 mark was reduced in both 13- and 22-month-old rat brain samples after ICH, with a greater reduction in 22-month-old rat brain samples (Figure 6E). Meanwhile, the H3K9ac mark increased to different levels in 13- and 22-month-old rat brain samples after ICH, and the 22-month-old rat brain samples showed a higher increase (Figure 6F). The H3K4me3 mark showed a global difference between 13- and 22-month-old rat brain samples and did not significantly change after ICH in either age group (Figure 6G). These results indicated that the gain of H3K9ac and/or H3K4me3 marks and loss of H3K27me3 may contribute to the activation of the IFN-γ signaling pathway and that these were involved in acute brain injury in aging ICH rats.

### Anti-IFN-γ Treatment Reduced Acute Brain Injury in ICH Rats

**Figure 7:**
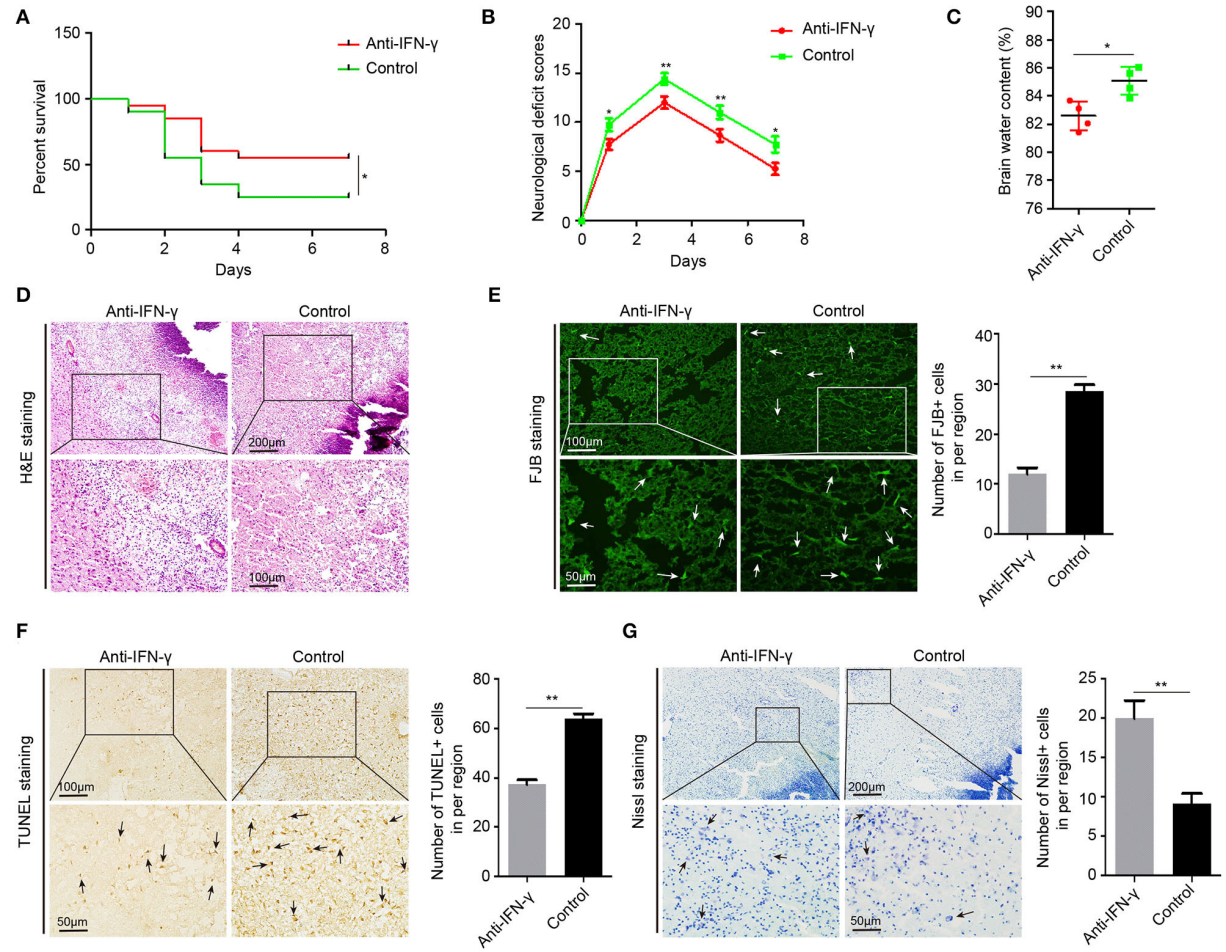
Anti-IFN-γ reduces acute brain injury in aging ICH rats. **(A)** The neurological deficit scores of 22-month-old ICH rats after treatment with anti-IFN-γ antibody or normal IgG (*n* = 12). **(B)** The mortality of aging ICH rats after treatment with anti-IFN-γ antibody or normal IgG (*n* = 20). **(C)** Level of brain water contents in perihematomal brain tissues in 22-month-old ICH rats 3 days after treatment with anti-IFN-γ antibody or normal IgG (*n* = 4). **(D)** Hematoxylin and eosin (H&E) staining of perihematomal brain tissue derived from 22-month-old ICH rats 3 days after treatment with anti-IFN-γ antibody or normal IgG. Scale bars, 200 μm at the top and 100 μm at the bottom. **(E)** Fluoro-Jade B (FJB) staining and quantification of degenerative neural cells in perihematomal brain tissues derived from 22-month-old ICH rats 3 days after treatment with an anti-IFN-γ antibody or normal IgG. Scale bars, 100 μm at the top and 50 μm at the bottom. **(F)** TUNEL assay and quantification of apoptotic neural cells in perihematomal brain tissues derived from 22-month-old ICH rats 3 days after treatment with anti-IFN-γ antibody or normal IgG. Scale bars, 100 μm at the top and 50 μm at the bottom. **(G)** Nissl staining and quantification of Nissl bodies in perihematomal brain tissues derived from 22-month-old ICH rats 3 days after treatment with anti-IFN-γ antibody or normal IgG. Scale bars, 200 μm at the top and 50 μm at the bottom. The bar graphs show the mean ± SEM. *p*-value determined using the Student's *t*-test. *p < 0.05, **p < 0.01, and ***p < 0.001.

To further confirm the effects of IFN-γ on acute brain injury, we injected neutralizing IFN-γ antibody in 22-month-old ICH rats through the tail vein 30 min after ICH surgery. In 22-month-old ICH rats, anti-IFN-γ treatment significantly reduced NDS, mortality, and BWC (Figures 7A–C), as well as ICH-induced edema, necrosis, and inflammatory cell infiltration around the hematoma (Figure 7D), and degenerative and apoptotic neurons (Figures 7E,F), but increased the number of neurons surviving around the hematoma (Figure 7G). Altogether, these data suggest that IFN-γ signaling contributes to acute brain injury response in aging ICH rats and anti-IFN-γ treatment may be a novel therapeutic approach in elderly ICH patients.

## Discussion

In this study, we reported differential reprogramming of the genome-wide profiles of three histone modifications H3K27me3, H3K4me3, and H4K9ac between 13- and 22-month-old ICH rat brains, which reflects the differential brain damage in these two populations of rats. In contrast, age had no significant effect on transcriptomic changes. This reprogramming study was carefully designed to compare the differences between the ipsilateral and contralateral regions of the brain in the same rats, eliminating the complications associated with genetic and epigenetic backgrounds in human studies. Thus far, few studies have been conducted on comprehensive epigenetic reprogramming. In fact, ICH-induced reprogramming of the genome-wide profile of any histone modification has not been reported yet. Previously, a number of studies have demonstrated the neuroprotection effects of histone deacetylase inhibitors in ICH mouse and rat models (Ren et al., 2004; Sinn et al., 2007; Sukumari-Ramesh et al., 2016), which indicate the involvement of histone modifications in response to ICH. The findings of this study provide new insights into age-dependent histone modifications induced by ICH.

Transcriptome studies related to ICH have been conducted using immune cells from peripheral blood of human participants (Dykstra-Aiello et al., 2015; Sang et al., 2017; Stamova et al., 2019), but changes in animal brain transcriptomes in response to ICH have not been reported. In this study, we used RNA-seq to profile the transcriptomic changes in ICH rat brains, revealing a prevalent gene activation and few gene inactivation events in the ipsilateral hemispheres of the brain. The activated genes function predominantly in immune/inflammatory responses. Interestingly, 13- and 22-month-old rat brains did not exhibit remarkably different transcriptomic changes (Figure 2), although the damage and recovery differed significantly (Figure 1). This finding may be expected because an array of inflammatory cells are either immediately activated or recruited in response to ICH-induced acute brain injury, which include resident immune cells of the brain (e.g., microglia and astrocytes) and infiltrating peripheral leukocytes (e.g., circulating macrophage and T helper cells). Damage-stimulated neuroinflammation reportedly causes secondary brain damage (Wang and Dore, 2007; Wang, 2010; Zhou et al., 2014; Zhang et al., 2017).

In this study, multiple histone marks associated with promoter regions were found to dynamically change in response to ICH. We investigated two activating marks, H3K4me3 and K4K9ac, and one repressive mark, H3K27me3. Strikingly, ICH-induced redistribution of H3K4me3 in 13-month-old rat brains was remarkably specific to inflammatory and immune response genes, whereas that of H3K27me3 was specific to neuroinflammation genes in 22-month-old rat brains. We further demonstrated that brain damage-induced DM dual histone marks are particularly specific to inflammatory and immune response genes in 22-month-old rats, whereas their specificity in 13-month-old rats is markedly less pronounced. These results have two implications. First, histone mark redistribution targets inflammatory and immune response genes in response to the brain damage caused by ICH. Second, the epigenomic response to ICH in 13- and 22-month-old rat brains markedly differed and was distinct from the transcriptomic response (Figure 2); this might represent a molecular mechanism underlying the substantial difference in ICH-induced damage between 13- and 22-month-old rats (Figure 1). These findings provide insights into the brain response to extracellular damage and may lead to the development of novel therapeutic strategies.

Chronic neuroinflammation is associated with a broad spectrum of age-related neurodegenerative diseases (McGeer and McGeer, 2004; Heppner et al., 2015; Murdock et al., 2015; Ransohoff, 2016). Aging enhances the susceptibility of individuals to neurodegenerative diseases (Ransohoff, 2016). However, the mechanistic links between chromatin structure and neuroinflammation remain obscure. The recently proposed concept of innate immune memory provides some mechanistic explanations (Netea et al., 2015). Two recent studies have highlighted the involvement of promoter-associated histone modification changes in establishing microglial innate immune memory, with one study showing a reduction in the activating histone modifications H3K9/K14 acetylation and H3K4 trimethylation in the promoters of the *IL-1*β and *TNF-*α genes (Schaafsma et al., 2015). Another study has shown complete loss of IL1β production, even after consecutive LPS challenges, in mice with a microglial-specific Hdac1/2 deletion (Wendeln et al., 2018). In line with the concept of innate immune memory, our findings demonstrated a systematic association of histone modification changes with the changed expression of many innate immune genes, including *IL-1*β, *STAT1*, and *IRF1*.

We further analyzed the correlation between promoter-associated histone mark redistribution and regulation of gene expression, demonstrating a strong correlation specific to ICH-induced but not aging-induced regulatory mechanisms. However, we could not dissect the functional relationship between histone mark redistribution and regulated expression of neuroinflammation-associated genes. It is unclear how histone mark redistribution affects gene expression changes and vice versa. Consistent with the concept of aging-regulated chromatin states (Sen et al., 2016), our results demonstrated substantially different histone mark profiles in 13- and 22-month-old rat brains. We demonstrated an age-dependent decrease of H3K27me3 and increase of H3K4me3 in rat brains, which is consistent with aging and longevity results obtained from experiments in worms (McColl et al., 2008; Jin et al., 2011; Maures et al., 2011).

We found that the cellular response to IFN-γ was significantly activated in ICH rats. IFN-γ is the only member of type II IFNs and is critical for both innate and adaptive immunity; it functions as a pro-inflammatory cytokine. In addition to its immunoregulatory activities, the role of IFN-γ in the regulation of brain functions is controversial (Baruch et al., 2014; Filiano et al., 2016; Papageorgiou et al., 2016; Chandrasekar et al., 2017; Sun et al., 2017). During the process of ICH in rodents, IFN-γ is often highly expressed. When IFN-γ is inhibited or downregulated, a significant improvement in neurological scores and a reduction in brain edema, neuroinflammation, and neurovascular injury are observed in mice/rodents with ICH (Rolland et al., 2013; Lu et al., 2014; Liu et al., 2016). Our current data indicate that the levels of IFN-γ in the brain are upregulated after ICH. Histone mark distributions around the TSSs of IFN-γ response genes were reprogrammed after ICH, suggesting that epigenetic reprogramming played an important role in the expression of IFN-γ response genes. Anti-IFN-γ antibody reduced acute brain injury in aging ICH rats, suggesting that an increase in brain IFN-γ levels led to the exacerbation of acute brain injury in aging ICH rats. This might be due to its role in the aggravation of neuroinflammatory response (Papageorgiou et al., 2016). In addition to the IFN-γ signaling pathway genes, there were many genes with increased expression levels in response to the ICH-induced acute brain injury, indicating that the brain injury-responsive factors require further study. Targeting one of these downstream regions of injury signaling pathways achieved certain therapeutic effects; hence, whether targeting the upstream regions of brain injury-responsive factors would achieve better curative effects requires further investigation.

Therefore, in this study, we demonstrated that ICH induced more pronounced histone mark redistribution in neuroinflammation-associated genes in late-aging rat brains, which could be determined by the different chromatin structures in 13- and 22-month-old rat brains. Further study of these age-regulated histone modifications and their specific responses to acute brain damage and other environmental stimuli may provide valuable information for understanding the mechanisms of age-sensitized neurodegenerative disease and for developing treatments against neuroinflammatory diseases and the neuroinflammatory state induced by acute brain damage.

## Data Availability Statement

The datasets presented in this study can be found in online repositories. The names of the repository/repositories and accession number(s) can be found in the article/Supplementary Material.

## Ethics Statement

Protocols of our animal studies were approved by and conducted in accordance with the Animal Ethics Committee of the Army Medical University (Third Military Medical University, Chongqing, China).

## Author Contributions

X-YX and Q-WY conceptualized the study, designed the experiments, and drafted this manuscript. KZ, RX, and LL established the ICH models for this study. QZ and W-lK performed anti-IFN-γ injection experiments. QZ and QC evaluated the NDS and mortality of ICH models. F-XW, J-CH, and G-QY conducted the staining and RNA-seq experiments. G-QY, J-CH, and QC performed the ELISA and ChIP-seq experiments. C-XG and QZ performed the PCR experiments. X-YX performed the RNA-seq and ChIP-seq data analysis. Q-WY supervised the study. All authors approved the final version of the manuscript.

## Funding

This study was supported in part by grants from the National Natural Science Fund for Distinguished Young Scholars (81525008).

## Conflict of Interest

The authors declare that the research was conducted in the absence of any commercial or financial relationships that could be construed as a potential conflict of interest.

## Publisher's Note

All claims expressed in this article are solely those of the authors and do not necessarily represent those of their affiliated organizations, or those of the publisher, the editors and the reviewers. Any product that may be evaluated in this article, or claim that may be made by its manufacturer, is not guaranteed or endorsed by the publisher.

## Supplementary Material

Supplementary Figure 1 Acute brain injury induced by ICH in rats. **(A)** Experimental diagram of ICH. **(B)** Hematoxylin and eosin (H&E) staining of perihematomal brain tissues derived from young and aging rats 3 days after ICH surgery. Scale bars, 200 μm at the top and 100 μm at the bottom. **(C)** Fluoro-Jade B (FJB) staining and quantification of degenerative neural cells in perihematomal brain tissues derived from young and aging rats 3 days after ICH. Scale bars, 100 μm at the top and 50 μm at the bottom. The bar graphs show the mean ± SEM; *p*-value was determined by Student's *t*-test. ^*^*p* < 0.05 and ^**^*p* < 0.01.

Supplementary Figure 2 Global RNA-seq profile and DEG functions in ICH-induced rat brain tissues. **(A)** Bar plot shows the genomic distribution of mapped reads from the first bench RNA-seq data. **(B)** Reads distribution profile of expressed transcripts in all sequenced samples from the first bench. Each transcript is divided into 100 parts from the transcription start site (TSS) to the transcription terminal site (TTS), and the read distribution of all transcripts is summarized. **(C)** Bubble plot shows the top 10 enriched GO BP terms of upregulated genes in 13-month Ipsi vs. control rats. **(D)** Bubble plot shows the top 10 enriched GO BP terms of upregulated genes in 22-month Ipsi vs. control rats.

Supplementary Figure 3 H3K27me3, H3K4me3, and H3K9ac changes in rat brain tissues after ICH. **(A)** Immunohistochemical staining of anti-H3K27me3, H3K4me3, and H3K9ac antibodies in the ipsilateral and contralateral brain tissues of young, adult, and old rats 3 days after ICH (*n* = 3). **(B)** Hierarchical clustering heatmap of all histone modification samples. PCCs between replicates were very high (>0.95). **(C)** Box plot shows the genomic distribution of DNA sequenced reads from ChIP-seq samples. **(D)** Line plot shows the distribution of Irreproducibility rate (IDR) by the number of significant peaks in the ChIP-seq samples for the three histone marks. **(E)** Reads density plot shows the distribution of three histone mark densities around two selected genes (*Stat1* and *Dapk1*). Gene structures are shown on the bottom track. ChIP-seq samples (the first replicate) of Ipsi and control in 13-month-old rats are shown in the figure.

Supplementary Figure 4 The distribution change of histone marks between Ipsi and control revealed their relevance to immune response genes. **(A)** Venn diagram shows the number of overlapping and specific peak clusters between Ipsi and control samples for H3K9ac in 13-month (left) and 22-month-old rats (right). **(B)** Bar plot shows the number of overlapping and specific genes between Ipsi and control samples for H3K9ac in 13-month (left) and 22-month-old rats (right). **(C,D)** Bar plot shows the top 10 enriched GO BP terms for genes with H3K9ac peaks in Ipsi samples of 13-month **(C)** and H3K9ac peaks in 22-month-old rats **(D)**. **(E)** Bubble plot shows the top 10 enriched GO BP terms of genes that have peaks in both Ipsi and control samples. Enriched terms of 13- and 22-month-old samples are shown with different colors. Three histone marks are shown separately.

Supplementary Figure 5 The pDMGs/rDMGs significantly overlapped with DEGs. **(A)** Upset bar plot shows the overlapping gene number among DEGs and three histone modified genes in 22-month Ipsi vs. 22-month Cont (left) and 22-month-Cont vs. 13-month-Cont (right) rats. **(B)** Hierarchical clustering heat map shows the expression level of genes that are differentially modified by histone marks in Ipsi of 22-month-old rat brains. **(C)** Bar plot shows the top 10 enriched GO BP terms of DEGs that overlap with DMGs (left) or do not overlap with DMGs (right) in 22-month-old ICH rats.

Supplementary Table 1 Summary of RNA-seq and ChIP-seq data.

Supplementary Table 2 The overlapped peaks between two biological replicates of ChIP-seq samples.

Supplementary Table 3 Summary of the peaks and genes analyzed by MACS2 software.

Supplementary Table 4 Primer sequences for qRT-PCR.
