# Supplementary figures and images for: Redistribution of Histone Marks on Inflammatory Genes Associated With Intracerebral Hemorrhage-Induced Acute Brain Injury in Aging Rats
Source: Front Neurosci. 2022 Apr 15;16:639656. doi: 10.3389/fnins.2022.639656 (PMC9051396; doi:10.3389/fnins.2022.639656)

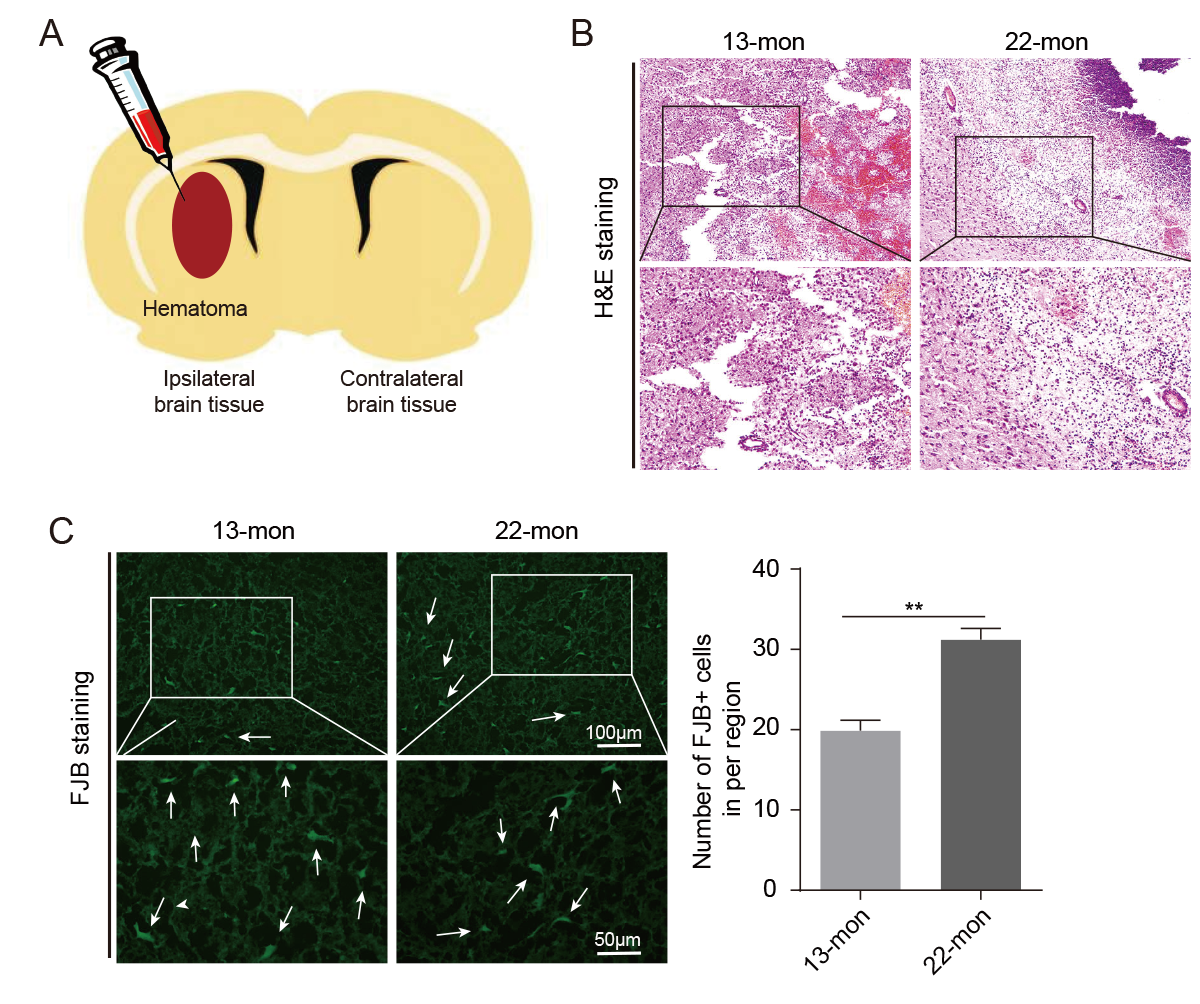

Supplement: Supplementary Figure 1 — Acute brain injury induced by ICH in rats. (A) Experimental diagram of ICH. (B) Hematoxylin and eosin (H&E) staining of perihematomal brain tissues derived from young and aging rats 3 days after ICH surgery. Scale bars, 200 μm at the top and 100 μm at the bottom. (C) Fluoro-Jade B (FJB) staining and quantification of degenerative neural cells in perihematomal brain tissues derived from young and aging rats 3 days after ICH. Scale bars, 100 μm at the top and 50 μm at the bottom. The bar graphs show the mean ± SEM; p-value was determined by Student's t-test. *p < 0.05 and **p < 0.01. [file Image_1.TIF]

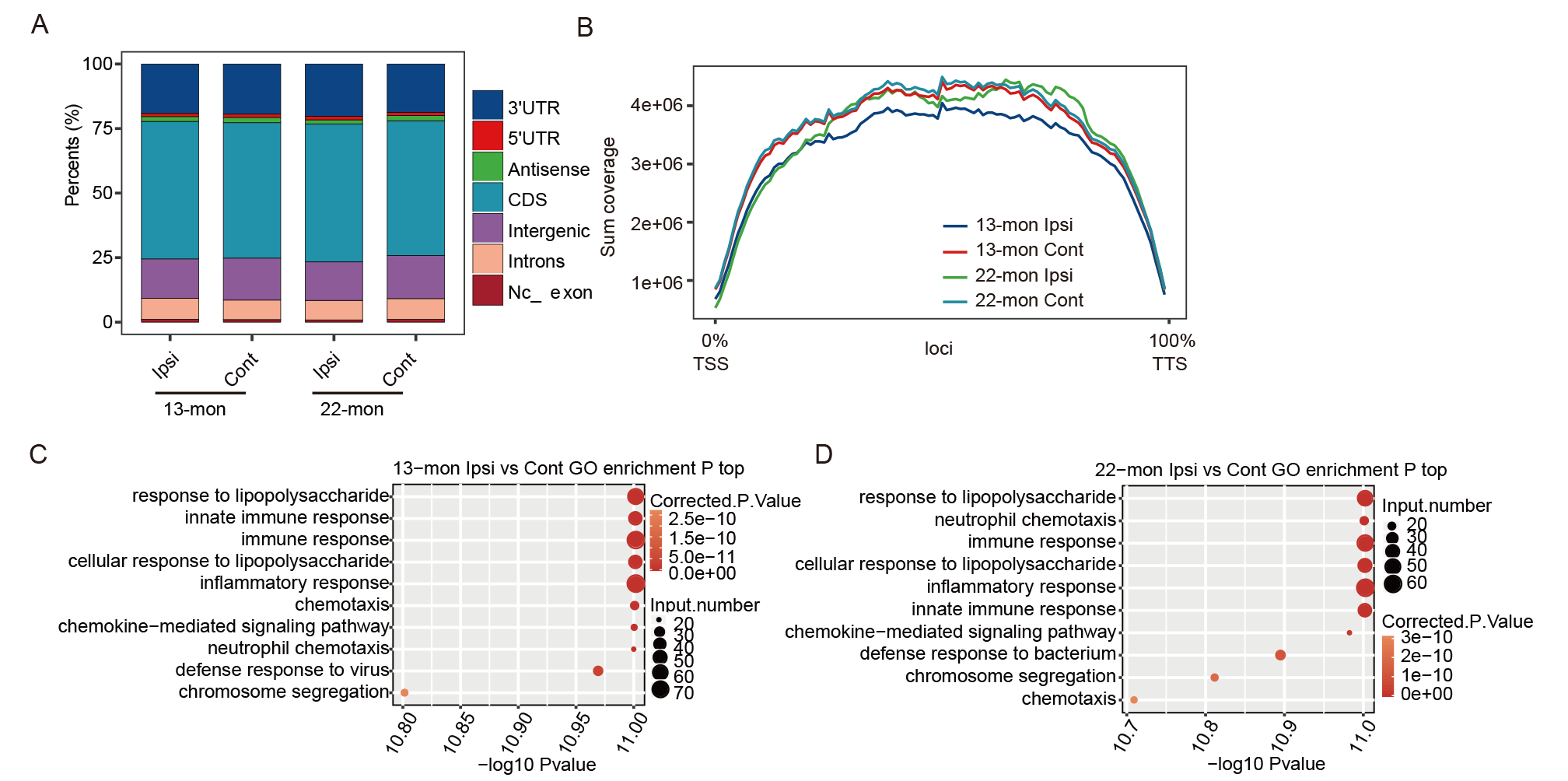

Supplement: Supplementary Figure 2 — Global RNA-seq profile and DEG functions in ICH-induced rat brain tissues. (A) Bar plot shows the genomic distribution of mapped reads from the first bench RNA-seq data. (B) Reads distribution profile of expressed transcripts in all sequenced samples from the first bench. Each transcript is divided into 100 parts from the transcription start site (TSS) to the transcription terminal site (TTS), and the read distribution of all transcripts is summarized. (C) Bubble plot shows the top 10 enriched GO BP terms of upregulated genes in 13-month Ipsi vs. control rats. (D) Bubble plot shows the top 10 enriched GO BP terms of upregulated genes in 22-month Ipsi vs. control rats. [file Image_2.TIF]

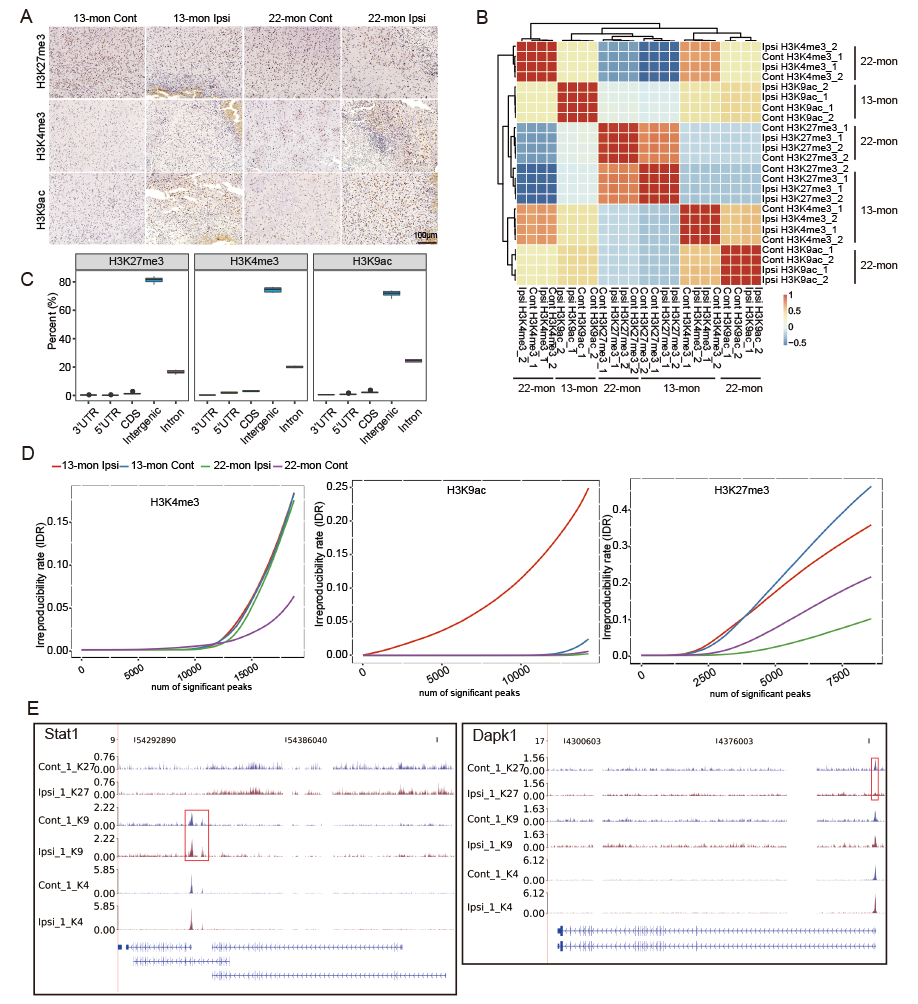

Supplement: Supplementary Figure 3 — H3K27me3, H3K4me3, and H3K9ac changes in rat brain tissues after ICH. (A) Immunohistochemical staining of anti-H3K27me3, H3K4me3, and H3K9ac antibodies in the ipsilateral and contralateral brain tissues of young, adult, and old rats 3 days after ICH (n = 3). (B) Hierarchical clustering heatmap of all histone modification samples. PCCs between replicates were very high (>0.95). (C) Box plot shows the genomic distribution of DNA sequenced reads from ChIP-seq samples. (D) Line plot shows the distribution of Irreproducibility rate (IDR) by the number of significant peaks in the ChIP-seq samples for the three histone marks. (E) Reads density plot shows the distribution of three histone mark densities around two selected genes (Stat1 and Dapk1). Gene structures are shown on the bottom track. ChIP-seq samples (the first replicate) of Ipsi and control in 13-month-old rats are shown in the figure. [file Image_3.TIF]

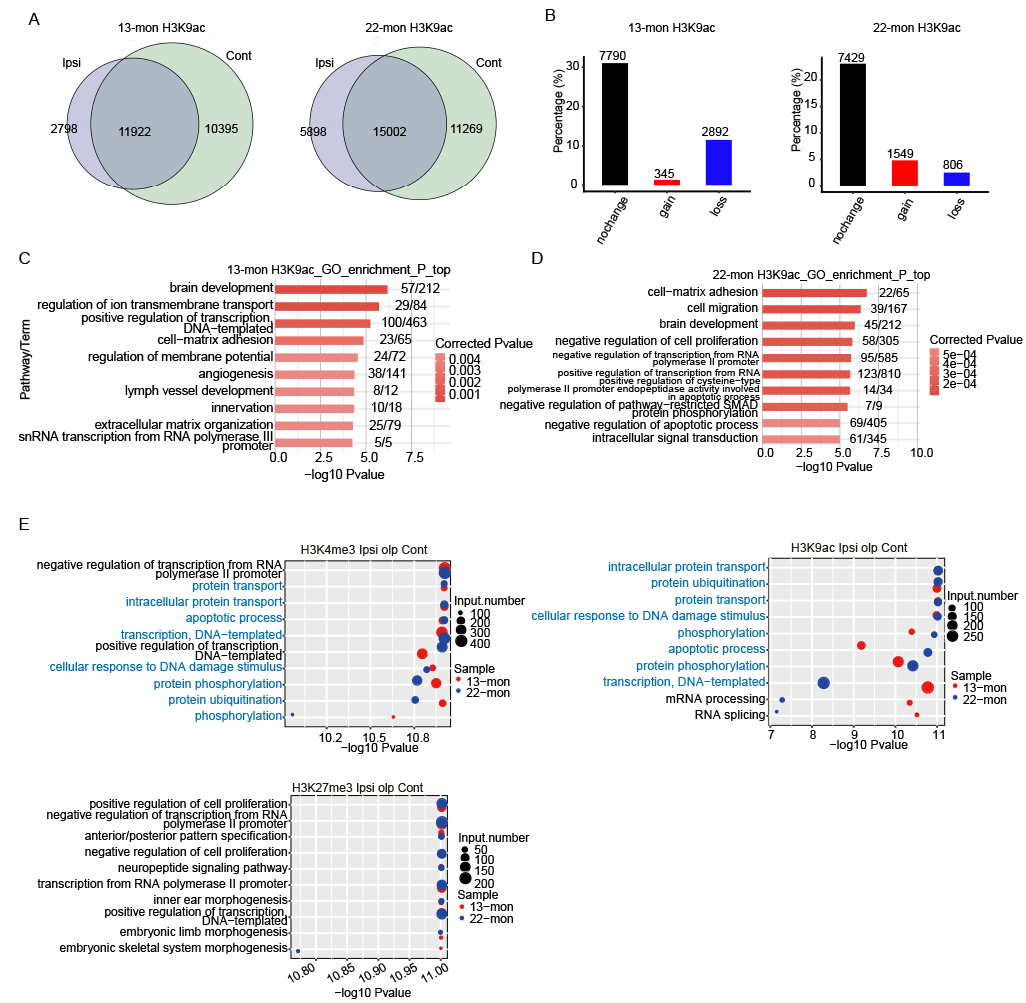

Supplement: Supplementary Figure 4 — The distribution change of histone marks between Ipsi and control revealed their relevance to immune response genes. (A) Venn diagram shows the number of overlapping and specific peak clusters between Ipsi and control samples for H3K9ac in 13-month (left) and 22-month-old rats (right). (B) Bar plot shows the number of overlapping and specific genes between Ipsi and control samples for H3K9ac in 13-month (left) and 22-month-old rats (right). (C,D) Bar plot shows the top 10 enriched GO BP terms for genes with H3K9ac peaks in Ipsi samples of 13-month (C) and H3K9ac peaks in 22-month-old rats (D). (E) Bubble plot shows the top 10 enriched GO BP terms of genes that have peaks in both Ipsi and control samples. Enriched terms of 13- and 22-month-old samples are shown with different colors. Three histone marks are shown separately. [file Image_4.TIF]

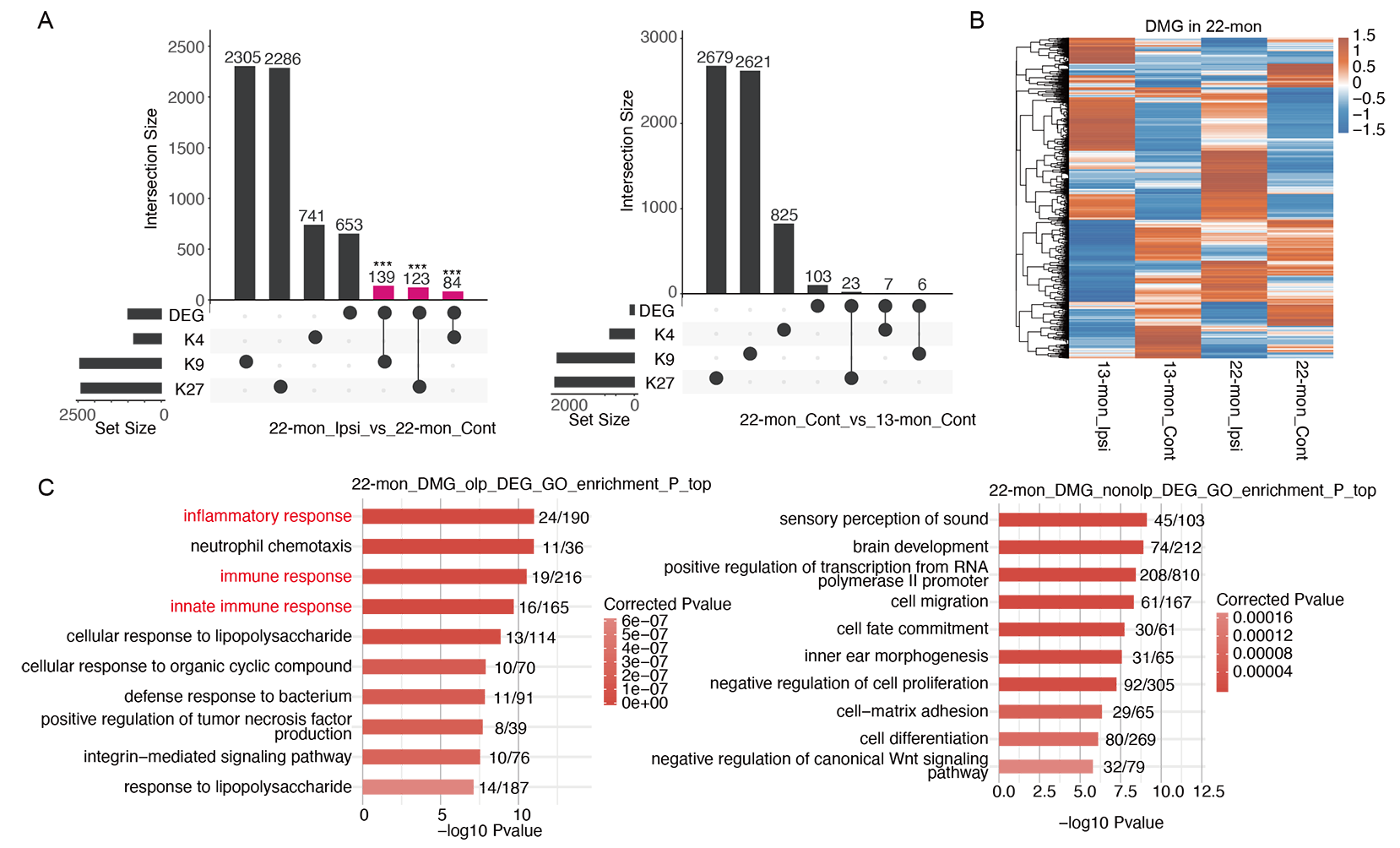

Supplement: Supplementary Figure 5 — The pDMGs/rDMGs significantly overlapped with DEGs. (A) Upset bar plot shows the overlapping gene number among DEGs and three histone modified genes in 22-month Ipsi vs. 22-month Cont (left) and 22-month-Cont vs. 13-month-Cont (right) rats. (B) Hierarchical clustering heat map shows the expression level of genes that are differentially modified by histone marks in Ipsi of 22-month-old rat brains. (C) Bar plot shows the top 10 enriched GO BP terms of DEGs that overlap with DMGs (left) or do not overlap with DMGs (right) in 22-month-old ICH rats. [file Image_5.TIF]
