# Supplementary material for: Redistribution of Histone Marks on Inflammatory Genes Associated With Intracerebral Hemorrhage-Induced Acute Brain Injury in Aging Rats
Source: Front Neurosci. 2022 Apr 15;16:639656. doi: 10.3389/fnins.2022.639656 (PMC9051396; doi:10.3389/fnins.2022.639656)
Supplement: Supplementary Table 1 — Summary of RNA-seq and ChIP-seq data. [file Table_1.DOCX]

**Supplementary Table S1** Summary of RNA-seq and ChIP-seq data.

| **Samples** | **Type** | **# of Raw reads** | **# of filtered reads** | **Filtered/Raw (%)** |
| --- | --- | --- | --- | --- |
| Adult Ipsi_1 | RNA-seq | 83574962 | 82061982 | 98.19 |
| Adult Ipsi_2 | RNA-seq | 81093770 | 79696926 | 98.28 |
| Adult Ipsi_3 | RNA-seq | 84316832 | 82702364 | 98.09 |
| Adult Cont_1 | RNA-seq | 81621484 | 80318360 | 98.40 |
| Adult Cont_2 | RNA-seq | 80399676 | 79030578 | 98.30 |
| Adult Cont_3 | RNA-seq | 65308974 | 64133876 | 98.20 |
| Old Ipsi_1 | RNA-seq | 85731918 | 84204932 | 98.22 |
| Old Ipsi_2 | RNA-seq | 75656958 | 74179482 | 98.05 |
| Old Ipsi_3 | RNA-seq | 83675386 | 82173706 | 98.21 |
| Old Cont_1 | RNA-seq | 79071892 | 77580780 | 98.11 |
| Old Cont_2 | RNA-seq | 126656024 | 124376646 | 98.20 |
| Old Cont_3 | RNA-seq | 72833902 | 71383880 | 98.01 |
| Adult Ipsi_H3K4me3_input_1 | ChIP-seq | 83134696 | 73637410 | 88.58 |
| Adult Ipsi _H3K4me3_1 | ChIP-seq | 85691452 | 76267622 | 89.00 |
| Adult Ipsi _H3K4me3_input_2 | ChIP-seq | 79212910 | 67745536 | 85.52 |
| Adult Ipsi _H3K4me3_2 | ChIP-seq | 89721370 | 78347483 | 87.32 |
| Adult Cont _H3K4me3_input_1 | ChIP-seq | 90290502 | 77702359 | 86.06 |
| Adult Cont _H3K4me3_1 | ChIP-seq | 93993632 | 82344593 | 87.61 |
| Adult Cont _H3K4me3_input_2 | ChIP-seq | 85246340 | 73354285 | 86.05 |
| Adult Cont _H3K4me3_2 | ChIP-seq | 93839068 | 82237631 | 87.64 |
| Old Ipsi _H3K4me3_input_1 | ChIP-seq | 76758198 | 64541034 | 84.08 |
| Old Ipsi _H3K4me3_1 | ChIP-seq | 79870844 | 69717726 | 87.29 |
| Old Ipsi _H3K4me3_input_2 | ChIP-seq | 84431488 | 71585675 | 84.79 |
| Old Ipsi _H3K4me3_2 | ChIP-seq | 79152226 | 67628122 | 85.44 |
| Old Cont_H3K4me3_input_1 | ChIP-seq | 88088840 | 75705740 | 85.94 |
| Old Cont_H3K4me3_1 | ChIP-seq | 97654056 | 84291383 | 86.32 |
| Old Cont_H3K4me3_input_2 | ChIP-seq | 87817132 | 75139195 | 85.56 |
| Old Cont_H3K4me3_2 | ChIP-seq | 86307810 | 73923628 | 85.65 |
| Adult Ipsi_H3K9ac_input_1 | ChIP-seq | 96910430 | 87298946 | 90.08 |
| Adult Ipsi_H3K9ac_input_2 | ChIP-seq | 146738756 | 130504102 | 88.94 |
| Adult Ipsi_H3K9ac_1 | ChIP-seq | 69765930 | 62666332 | 89.82 |
| Adult Ipsi_H3K9ac_2 | ChIP-seq | 71631574 | 64771582 | 90.42 |
| Adult Cont_H3K9ac_input_1 | ChIP-seq | 98347632 | 88160496 | 89.64 |
| Adult Cont_H3K9ac_input_2 | ChIP-seq | 66405168 | 58741214 | 88.46 |
| Adult Cont_H3K9ac_1 | ChIP-seq | 70528422 | 63116657 | 89.49 |
| Adult Cont_H3K9ac_2 | ChIP-seq | 72085714 | 64823576 | 89.93 |
| Old Ipsi_H3K9ac_input_1 | ChIP-seq | 76769708 | 63593734 | 82.84 |
| Old Ipsi_H3K9ac_1 | ChIP-seq | 68297204 | 55849991 | 81.77 |
| Old Ipsi_H3K9ac_input_2 | ChIP-seq | 67882190 | 55620931 | 81.94 |
| Old Ipsi_H3K9ac_2 | ChIP-seq | 67025938 | 55279541 | 82.47 |
| Old Cont_H3K9ac_input_1 | ChIP-seq | 70787428 | 58545814 | 82.71 |
| Old Cont_H3K9ac_1 | ChIP-seq | 76929000 | 65455772 | 85.09 |
| Old Cont_H3K9ac_input_2 | ChIP-seq | 82795886 | 69772783 | 84.27 |
| Old Cont_H3K9ac_2 | ChIP-seq | 79862718 | 67861043 | 84.97 |
| Adult Cont_H3K27me3_input_1 | ChIP-seq | 78189808 | 67365676 | 86.16 |
| Adult Cont_H3K27me3_1 | ChIP-seq | 74624852 | 64475307 | 86.40 |
| Adult Cont_H3K27me3_input_2 | ChIP-seq | 88716600 | 76590830 | 86.33 |
| Adult Cont_H3K27me3_2 | ChIP-seq | 69101534 | 59058865 | 85.47 |
| Adult Ipsi_H3K27me3_input_1 | ChIP-seq | 110700172 | 95034429 | 85.85 |
| Adult Ipsi_H3K27me3_1 | ChIP-seq | 75039022 | 64826194 | 86.39 |
| Adult Ipsi_H3K27me3_input_2 | ChIP-seq | 127408234 | 113112944 | 88.78 |
| Adult Ipsi_H3K27me3_2 | ChIP-seq | 73232850 | 63509275 | 86.72 |
| Old Cont_H3K27me3_input_1 | ChIP-seq | 107974378 | 94090333 | 87.14 |
| Old Cont_H3K27me3_input_2 | ChIP-seq | 103104826 | 90615536 | 87.89 |
| Old Cont_H3K27me3_1 | ChIP-seq | 97066056 | 81857738 | 84.33 |
| Old Cont_H3K27me3_2 | ChIP-seq | 88856500 | 75835598 | 85.35 |
| Old Ipsi_H3K27me3_input_1 | ChIP-seq | 105376964 | 92993697 | 88.25 |
| Old Ipsi_H3K27me3_input_2 | ChIP-seq | 103193912 | 90120749 | 87.33 |
| Old Ipsi_H3K27me3_1 | ChIP-seq | 112669124 | 101459596 | 90.05 |
| Old Ipsi_H3K27me3_2 | ChIP-seq | 98704816 | 87792549 | 88.94 |

**Supplementary Table S2** The overlapped peaks between two biological replicates of ChIP-seq samples.

| Histone Mark | Sample | Peaks | Overlapped Peaks |
| --- | --- | --- | --- |
| H3K4me3 | m13_ICH_H3K4Me3_1 | 20548 | 16716 |
|  | m13_ICH_H3K4Me3_2 | 18401 |  |
|  | m13_Cont_H3K4Me3_1 | 18676 | 16204 |
|  | m13_Cont_H3K4Me3_2 | 18694 |  |
|  | m22_ICH_H3K4me3_1 | 19274 | 17167 |
|  | m22_ICH_H3K4me3_2 | 20114 |  |
|  | m22_Cont_H3K4me3_1 | 22201 | 19692 |
|  | m22_Cont_H3K4me3_2 | 23500 |  |
| H3K9ac | 13m_ICH_H3K9ac_2 | 45691 | 15066 |
|  | 13m_ICH_H3K9ac_1 | 50966 |  |
|  | 13m_Cont_H3K9ac_1 | 48101 | 22664 |
|  | 13m_Cont_H3K9ac_2 | 30847 |  |
|  | m22_ICH_H3K9ac_1 | 31510 | 21630 |
|  | m22_ICH_H3K9ac_2 | 44009 |  |
|  | m22_Cont_H3K9ac_2 | 44131 | 26541 |
|  | m22_Cont_H3K9ac_1 | 40402 |  |
| H3K27me3 | m13_ICH_H3K27Me3_2 | 54588 | 11393 |
|  | m13_ICH_H3K27Me3_1 | 24637 |  |
|  | m13_Cont_H3K27Me3_1 | 19822 | 7819 |
|  | m13_Cont_H3K27Me3_2 | 44530 |  |
|  | 22m_Cont_H3K27me3_2 | 155145 | 32609 |
|  | 22m_Cont_H3K27me3_1 | 59812 |  |
|  | 22m_ICH_H3K27me3_1 | 79270 | 46509 |
|  | 22m_ICH_H3K27me3_2 | 106446 |  |

**Supplementary Table S3.** Summary of the peaks and genes analyzed by MACS2 software.

| **Marker** | **Sample** | **Peaks** | **TSS5k4fe Peak** | **TSS5k4fe Gene** | **Specific Peak** | **Specific Gene** |
| --- | --- | --- | --- | --- | --- | --- |
| H3K27me3 | Adult Ipsi | 117866 | 2859 | 2014 | 1607 | 1403 |
|  | Adult Cont | 51805 | 4741 | 3073 |  |  |
|  | Old Ipsi | 300668 | 4420 | 3008 | 4104 | 3080 |
|  | Old Cont | 367061 | 9743 | 5840 |  |  |
| H3K4me3 | Adult Ipsi | 33661 | 14293 | 11720 | 572 | 526 |
|  | Adult Cont | 33574 | 14169 | 11430 |  |  |
|  | Old Ipsi | 30356 | 14469 | 11939 | 148 | 127 |
|  | Old Cont | 35063 | 15237 | 12529 |  |  |
| H3K9ac | Adult Ipsi | 69557 | 20905 | 12403 | 1204 | 949 |
|  | Adult Cont | 93770 | 16627 | 11945 |  |  |
|  | Old Ipsi | 102607 | 15349 | 11454 | 2919 | 2505 |
|  | Old Cont | 120348 | 12973 | 9308 |  |  |

Note: Specific Peak and gene for H3K27me3 indicates peaks and genes specific for Cont compared with Ipsi. Specific Peak and gene for H3K4me3 and H3K9ac indicate the peaks and genes specific for Ipsi compared with Cont. Ipsi, the ipsilateral brain tissue, Cont, the contralateral brain tissue, TSS, transcription start site. The peaks were enriched 4-folds that localized within 5kb from TSS.

**Supplementary Table 4.** Primer sequences for qRT-PCR.

| **Gene name** | **Primer sequences (forward and reverse)** |
| --- | --- |
| Aif1 | 5'-TGTGTTGGCTGGCTCCTCT-3' and 5'-ACTTGGTCCGCCTTCTCCT-3' |
| Gbp5 | 5'-GAGAGGCAGAGACACAGAGA-3' and 5'-CCATCAATCTCTCAATCCCTCT-3' |
| RT1-Bb | 5'-GGGAACACGGAGCAGAGTT-3' and 5'-ACAGGGCAAAGGTGAGGAAG-3' |
| Cebpa | 5'-GCGGCGGTGACTTTGACT-3' and 5'-AGCCTGCCGTCCAGGTAG-3' |
| Ccl6 | 5'-CCCACTAAGAACAATGGAAACA-3' and 5'-ATACTGGCATTCGCTTCTCATA-3' |
| Ccl4 | 5'-TGGAAGATTCTCAAGTCTGTTG-3' and 5'-CCTCTATTGGCTGCTAATTCTT-3' |
| Ccr7 | 5'-AATTGAGTGGTTCAGGTGACGT-3' and 5'-AGAGAGGGCTGTGGGTGTGT-3' |
| Gbp2 | 5'-GCTATTACCTGCCAAATACCAC-3' and 5'-AGAAAGCCTGCATAATATCCAG-3' |
| Cd8a | 5'-CAATGCCTCTTCCTCACCAA-3' and 5'-GGTTAGCCTAAGCTACCTACTA-3' |
| Fgr | 5'-CCGACGGAAATCCTGCTTT-3' and 5'-ACGAAGGCGGGAACCAAA-3' |
| Uhrf1 | 5'-TAGATATAGCCCGCAGGGTAAG-3' and 5'-GGGAAGCGAGAAAGCAAGTG-3' |
| Lrg1 | 5'-TGTGGCAACTAGGGATTCAAAC-3' and 5'-GAGGCAGAGGCAGAAGAAGA-3' |
| Plin5 | 5'-GCTGTGCCCTAATGTAACTGTT-3' and 5'-CCTGTCCTTGGCTGAGTTGT-3' |
| Ifng | 5'-AAGTTCTCAGGCTTTGATGGC-3' and 5'-AGTTAGAGGCAGACTCAGGTAG-3' |
| LOC498435 | 5'-TGTAGATTGCGGTCCTGCC-3' and 5'-CGGTGAGAAACAGAGTCTTAGG-3' |
| LOC685067 | 5'-AAGCCCATTTCACATTAATTTCA-3' and 5'-TGTTATCTTTAGCAATGGGACTT-3' |
| Gbp4 | 5'-CAGGCATAGTCACAAGAGTAAGG-3' and 5'-TGTTATCTTTAGCAATGGGACTT-3' |
| Ccl5 | 5'-AAGGCAGCAAGGAGCTACAA-3' and 5'-AAACCGGGCAGCCTTTGGT-3' |
